# Signaling protein signature predicts clinical outcome of non-small-cell lung cancer

**DOI:** 10.1186/s12885-018-4104-4

**Published:** 2018-03-06

**Authors:** Bao-Feng Jin, Fan Yang, Xiao-Min Ying, Lin Gong, Shuo-Feng Hu, Qing Zhao, Yi-Da Liao, Ke-Zhong Chen, Teng Li, Yan-Hong Tai, Yuan Cao, Xiao Li, Yan Huang, Xiao-Yan Zhan, Xuan-He Qin, Jin Wu, Shuai Chen, Sai-Sai Guo, Yu-Cheng Zhang, Jing Chen, Dan-Hua Shen, Kun-Kun Sun, Lu Chen, Wei-Hua Li, Ai-Ling Li, Na Wang, Qing Xia, Jun Wang, Tao Zhou

**Affiliations:** 1 State Key Laboratory of Proteomics, Institute of Basic Medical Sciences, China National Center of Biomedical Analysis, Beijing, 100850 China; 2 Department of Thoracic Surgery, People’s Hospital, Peking University, Beijing, 100044 China; 3 Computational Medicine Laboratory, Beijing Institute of Basic Medical Sciences, Beijing, 100850 China; 4 The 90th Hospital of Jinan, Jinan, 250031 China; 5 Department of Pathology, People’s Hospital, Peking University, Beijing, 100044 China; 6 Institute of Pathology, Southwest Cancer Center, Southwest Hospital, Chongqing, 400038 China; 7 Department of Pathology, The 307th Hospital of Chinese PLA, Beijing, 100071 China

**Keywords:** Adenocarcinoma, Non-small-cell lung cancer, Prognosis, Protein signature, Squamous cell carcinoma

## Abstract

**Background:**

Non-small-cell lung cancer (NSCLC) is characterized by abnormalities of numerous signaling proteins that play pivotal roles in cancer development and progression. Many of these proteins have been reported to be correlated with clinical outcomes of NSCLC. However, none of them could provide adequate accuracy of prognosis prediction in clinical application.

**Methods:**

A total of 384 resected NSCLC specimens from two hospitals in Beijing (BJ) and Chongqing (CQ) were collected. Using immunohistochemistry (IHC) staining on stored formalin-fixed paraffin-embedded (FFPE) surgical samples, we examined the expression levels of 75 critical proteins on BJ samples. Random forest algorithm (RFA) and support vector machines (SVM) computation were applied to identify protein signatures on 2/3 randomly assigned BJ samples. The identified signatures were tested on the remaining BJ samples, and were further validated with CQ independent cohort.

**Results:**

A 6-protein signature for adenocarcinoma (ADC) and a 5-protein signature for squamous cell carcinoma (SCC) were identified from training sets and tested in testing sets. In independent validation with CQ cohort, patients can also be divided into high- and low-risk groups with significantly different median overall survivals by Kaplan-Meier analysis, both in ADC (31 months vs. 87 months, HR 2.81; *P* <  0.001) and SCC patients (27 months vs. not reached, HR 9.97; *P* <  0.001). Cox regression analysis showed that both signatures are independent prognostic indicators and outperformed TNM staging (ADC: adjusted HR 3.07 vs. 2.43, SCC: adjusted HR 7.84 vs. 2.24). Particularly, we found that only the ADC patients in high-risk group significantly benefited from adjuvant chemotherapy (*P* = 0.018).

**Conclusions:**

Both ADC and SCC protein signatures could effectively stratify the prognosis of NSCLC patients, and may support patient selection for adjuvant chemotherapy.

**Electronic supplementary material:**

The online version of this article (10.1186/s12885-018-4104-4) contains supplementary material, which is available to authorized users.

## Background

Lung cancer is the most common cause of cancer-related mortality worldwide, and approximately 80% cases are non-small-cell lung cancer (NSCLC) mainly including adenocarcinomas (ADC) and squamous cell carcinomas (SCC) [1, 2]. The tumor-node-metastasis (TNM) staging system is currently adopted to predict prognosis and guide treatment decisions for patients with NSCLC [3, 4]. However, the current staging system is not always accurate [5, 6]. After complete surgical resection of NSCLC, about 30% pathologic stage IA patients die of relapse within five years, while nearly 50% of stage IIA and 24% stage IIIA patients can survive [6].

To improve the outcome prediction of NSCLC, tremendous efforts have been made to identify prognostic markers. Based on gene microarray analysis, many groups have identified different gene signatures [7–19]. These signatures were usually identified on fresh or frozen tissue samples, the mixture of stroma, tumor and normal cells. In addition, mRNA levels in gene-based signatures are not always consistent with protein expression levels. Until now, this approach has not yielded a signature that could be applied in NSCLC clinical practice [13]. On the other hand, the management of cancer patients is still mainly guided based on combinations of clinicopathological features, including prognostic markers derived from careful histopathological analysis of tumors. For breast cancer, the combination of three protein markers, oestrogen receptor (ER), progesterone receptor (PR) and human epidermal growth factor receptor 2 (HER2), has been successfully utilized for clinical decision making and the use of this framework has contributed to the steady decline in the mortality of breast cancer patients. For lung cancer, numerous signaling proteins have been identified as abnormal in cancer development and progression [1, 20–22], and many of them have been reported to be correlated with clinical outcome of NSCLC [23]. However, none of them could provide adequate accuracy in clinical application. Based on these proteins, we attempt to identify a multi-protein signature to improve the prognosis prediction of NSCLC.

In this study, we performed an immunohistochemistry (IHC) analysis of 75 signaling proteins in paraffin-embedded surgical specimens of NSCLCs. These proteins represent the most important signaling pathways involved in cancer development [20, 21, 24]. Using random forest algorithm (RFA) and support vector machines (SVM), we successfully identified a 6-protein signature for ADC and a 5-protein signature for SCC that accurately predicted prognosis of ADC and SCC respectively.

## Methods

### Study design

The objective of this study was to identify signaling protein signature for NSCLC prognosis. Patients were eligible to enter the study if they underwent complete resection of invasive NSCLC at People’s Hospital of Peking University in Beijing (BJ) and Southwest Hospital of Chongqing (CQ) between January, 2004, and December, 2010. Information on the clinical variables and follow-up data were obtained from a prospectively maintained database of individual hospitals. We then excluded the patients who received any treatment prior to resection, received epidermal growth factor receptor tyrosine kinase inhibitor (EGFR TKI), died within 30 days of resection, or had no follow-up information. In total, 211 samples of patients from BJ hospital and 173 samples of patients from CQ hospital who fulfilled the inclusion criteria were collected for our study. The protocols of this study were approved by the Institutional Review Board of People’s Hospital and Southwest Hospital. Written consent for the use of the resected tissue for research purposes was obtained at time of surgery.

We assessed the expression levels of 75 signaling proteins via immunohistochemistry (IHC) on BJ samples. BJ samples were randomly partitioned into training (2/3 samples) and testing (1/3 samples) sets. Random forest algorithm and support vector machines were employed to identify prognostic signatures. Since previous studies have suggested fundamental differences between ADC and SCC regarding their molecular make-ups [2, 25], we separately identified the signature for ADC and SCC patients. CQ cohort was used for independent validation.

### Selection of IHC markers

To create the panel of IHC markers, we started with all of the 1027 proteins in “pathways in cancer” (Entry No. map05200) and “non-small cell lung cancer” (Entry No. map05223) of KEGG (Kyoto Encyclopedia of Genes and Genomes) pathway database. After searching NCBI Gene database using gene IDs and the related articles in PubMed (“See all citations in PubMed” under item “Bibliography” in the webpages), we found that 765 of 1027 candidates were associated with prognosis of cancer. After further filtering with “IHC” and “FFPE” and checking the existing literature, we found that 103 of the 765 proteins had been tested with IHC assay on FFPE samples in prognosis analysis.

We then purchased antibodies against all these 103 proteins and tested them by Western blot and immunofluorescence analysis. Seventy-four antibodies against 74 different proteins/phospho-proteins were found with high specificity/sensitivity and were used in our subsequent study. These 74 proteins/phospho-proteins plus CUEDC2, a potential oncogene that we identified and studied extensively [26–29], were the final members of the IHC marker panel. Schematic diagram of IHC marker selection is depicted in Additional file 1: Figure S1.

### Tissue microarray

All hematoxylin and eosin (H&E) slides were centrally reviewed at Department of Pathology in People’s Hospital according to the histopathological classification system adopted by the World Health Organization (WHO) to confirm tumor type and differentiation grade. Tissue microarrays were prepared as previously described [30]. Representative areas of each tissue sample were identified and carefully marked on H&E-stained sections. Core-tissue specimens (2 mm in diameter) were punched from the corresponding individual donor tissue blocks and rearranged in recipient blocks using a trephine apparatus (SuperBioChips Laboratories, Seoul, Korea) [31].

### IHC analysis

IHC staining was performed and evaluated as previously described [32]. The consecutive 4-μm-thick sections of tissue array were cut and mounted on glass slides. The slides were baked at 60 °C for 2 h prior to the high-throughput IHC procedure. The arrays were deparaffinized via sequential washing with xylene, graded ethanol and water. Antigens were retrieved (or not) for 15 min at 95 °C (see Additional file 1: Table S1 for detail). Endogenous peroxidase was blocked with 3% H_2_O_2_ for 30 min. Nonspecific staining was blocked using 10% normal goat serum (in 1× PBS) for 1 h at room temperature. The slides were incubated overnight at 4 °C with various antibodies (diluted in 1× PBS, see Additional file 1: Table S1 for dilution). The enhancing step, incubation with the secondary antibody (1 h at room temperature) and the diaminobenzidine (DAB) substrate (5 min at room temperature) were all performed following the protocol of ABC kit (Vector Laboratories, Burlingame, CA). Hematoxylin was used as a counterstain in the last step. The slides were then rinsed, cleared and mounted. The staining of each antibody was optimized based on negative and positive controls. For complete data of optimal antibody dilutions and assay conditions, see Additional file 1: Table S1. The flurescent images and Westhern blot results for specificity/sensitivity validation of each antibody are deposited in Clinical Research Database (CRD, see http://202.38.152.246:81/crd2/frontend/www/, username: nsclc, password: nsclcnsclc).

Semiquantative analysis of the immunostained slides was performed using a modified histochemical scoring (H-score) system to assess both the intensity of the staining and the percentage of positively-stained cells, as previously described [32]. Briefly, for the intensity, a score of 0 to 3 (corresponding to negative, weak, moderate or strong staining) was recorded. The scoring was normalized with controls. In addition, the percentage of positively-stained cells at each intensity was estimated. The H-score was calculated as 1 × (weak %) + 2 × (moderate %) + 3 × (strongly stained %). All slides were concurrently evaluated by three certified pathologists (Y.H.T., D.H.S and K.K.S), blinded to clinical data, to improve the accuracy of the results. The evaluation was repeated under multi-headed microscope if there was a discrepancy between the pathologists in the interpretation of the slides until consensus was achieved. All the digital images and the results of IHC evaluation are deposited in CRD.

### Data process of protein expression profiles

The expression score of each protein was processed for further analysis. Missing values were replaced with the median score of the respective protein in all tumors (see Supplementary methods for the details). The ratio of the expression score of each protein in a single sample to the mean score of that protein was calculated. The expression level was then quantified as log_10_ (expression ratio). To avoid zeros in the logarithm, a score of 0.01 was added to all scores. For independent validation, missing values were replaced with the median score. The expression profiles were processed as described above.

### Signature identification and model development

The patients were grouped according to the survival status at three years for modeling. Two-thirds of ADC and SCC patients from BJ cohort were assigned as training sets by computer-generated random numbers. Random forest algorithm was used to identify protein signatures in the training sets [33]. The procedure was implemented using the R varSelRF package with parameters “ntree = 5000, ntreeIterat = 2000, vars.drop.frac = 0.2”, which was built upon the randomForest package [34, 35]. The set of proteins with the smallest out-of-bag error rate among all the forests were returned and selected as signatures.

After signatures were identified, SVM was employed to develop the classification models in the training sets. The radial basis function (RBF) kernel was chosen for SVM training. The parameters C and γ for RBF kernel were tuned using the grid search strategy [36]. The parameter C was tuned from 2^− 5^ to 2^15^ with the step of 2^2^. The parameter γ was tuned from 2^3^ to 2^− 15^ with the step of 2^− 2^. During the training phase, the performance of SVM was evaluated using 5-fold cross-validation accuracy. The classification model was trained using SVM with the optimal C and γ. All the procedure was implemented using libSVM, a library for support vector machines [36].

To analyze the robustness of our signatures, enrichment analysis was performed. Ten thousand training sets were generated from BJ ADC and BJ SCC patients respectively, by randomly partitioning BJ ADC and BJ SCC patients into training set and testing set for 10,000 times. Each training set involved 2/3 ADC or SCC patients. A signature was identified on each training set using random forest algorithm. For each protein, the fraction of the signatures containing the protein (i.e. percentage of subsets) in all 10,000 subsets in ADC or SCC patients from BJ cohort was calculated. The proteins were sorted in descending order of the percentages.

### Statistical analysis

Overall survival (OS) from the time of resection was chosen as the primary endpoint since it is verifiable through multiple sources and less subject to interpretation bias [14]. Differences in survival between patients with good and poor prognosis and between patients treated with adjuvant chemotherapy or not were analyzed using Kaplan-Meier analysis and the two-sided log-rank test. Related covariates in this study were compared with clinical outcome using univariate and multivariate Cox regression analysis. A Wald likelihood ratio test was performed to assess statistical significance. For all statistical tests, a two-sided α of 0.05 was regarded as statistically significant. The analyses were performed in the R programming language (version 3.0.2).

## Results

### Patient characteristics

In this study, 211 patients who fulfilled the inclusion criteria from Beijing People’s Hospital were used as the training and testing sets (BJ cohort) (Table 1). A total of 173 patients from Chongqing Southwest Hospital were used as the independent validation cohort (CQ cohort) (Table 1). All the samples are resected tissues from patients underwent surgery at TNM stages I-IIIA. Of the total cases, 63% (243 of 384) patients received adjuvant platinum-based doublet chemotherapy. The median duration of follow-up was 58 months (interquartile range, 22 to 75), and during the follow-up period, 175 patients died (Table 1). Because previous studies have suggested fundamental differences between ADC and SCC regarding their molecular make-ups [2, 25], we separately identified the prognostic signature for ADC and SCC patients based on the expression levels of the detected signaling proteins.

**Table 1:** Clinical-pathological characteristics of non-small-cell lung cancer patients

Variable	All patients (*n* = 384)	BJ cohort (*n* = 211)	CQ cohort (*n* = 173)
Age			
≤60	151 (39.32%)	73 (34.60%)	78 (45.09%)
> 60	233 (60.68%)	138 (65.40%)	95 (54.91%)
Gender			
Male	270 (70.31%)	135 (63.98%)	135 (78.03%)
Female	114 (29.69%)	76 (36.02%)	38(21.97%)
Smoking index (pack years)			
Never smokers	142 (36.98%)	94 (44.55%)	48 (27.75%)
Light smokers (< 20)	41 (10.68%)	26 (12.32%)	15 (8.67%)
Heavy smokers (≥20)	183 (47.65%)	88 (41.71%)	95 (54.91%)
Unknown	18 (4.69%)	3 (1.42%)	15 (8.67%)
Histology			
Adenocarcinoma	206 (53.65%)	122 (57.82%)	84 (48.55%)
Squamous cell carcinoma	178 (46.35%)	89 (42.18%)	89 (51.45%)
Follow-up (months; median; IQR)	58 (22–75)	59 (19–74)	56 (29–76)
Deaths	175 (45.57%)	93 (44.07%)	82 (47.40%)
Differentiation grade			
Well	75 (19.53%)	45 (21.33%)	30 (17.34%)
Moderate	140 (36.46%)	67 (31.75%)	73 (42.20%)
Poor	138 (35.94%)	98 (46.45%)	40 (23.12%)
Unknown	31 (8.07%)	1 (0.47%)	30 (17.34%)
Pathologic stage			
I	188 (48.96%)	95 (45.02%)	93 (53.76%)
II	101 (26.30%)	47 (22.28%)	54 (31.21%)
III	95 (24.74%)	69 (32.70%)	26 (15.03%)
Adjuvant chemotherapy			
No	141 (36.72%)	100 (47.39%)	41 (23.70%)
Yes	243 (63.28%)	111 (52.61%)	132 (76.30%)

Data are number (%), unless otherwise stated. IQR = interquantile range

### Identification of the ADC signature

**Fig. 1:**
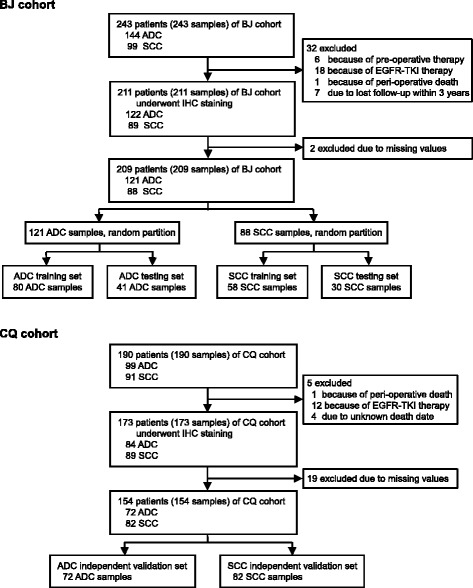
Flow chart of signature identification and validation strategy. Abbreviations: BJ cohort, patients from Peking University People’s Hospital; CQ cohort, patients from Southwest Hospital of Chongqing; ADC, adenocarcinoma; SCC, squamous cell carcinoma; IHC, immunohistochemistry; EGFR-TKI, epidermal growth factor receptor tyrosine kinase inhibitor

In our experiments, all the antibodies used were commercially available and were validated by Western blot and immunofluorescence analysis (Additional file 1: Table S1). Using IHC, we investigated the expression levels of 75 signature protein candidates in BJ cohort (Additional file 1: Table S1). To make the detailed staining and clinical information of each case accessible to the public, we developed a clinical research database (CRD) and all the results were deposited in it (http://202.38.152.246:81/crd2/frontend/www/, username: nsclc, password: nsclcnsclc). Two thirds of ADC patients from BJ cohort were randomly assigned as the training set, and the remaining 1/3 as the testing set (Fig. 1, BJ cohort). We performed signature discovery using random forest algorithm on the training set. The identified signature of ADC comprised six proteins: c-SRC, Cyclin E1, TTF1, p65, CHK1, and JNK1. We developed a classification model with the 6-protein signature using SVM algorithm (Additional file 1: Tables S2 and S3). For each patient, the model was used to calculate a prognosis score, which represents the combined information of the six proteins in the signature.

**Fig. 2:**
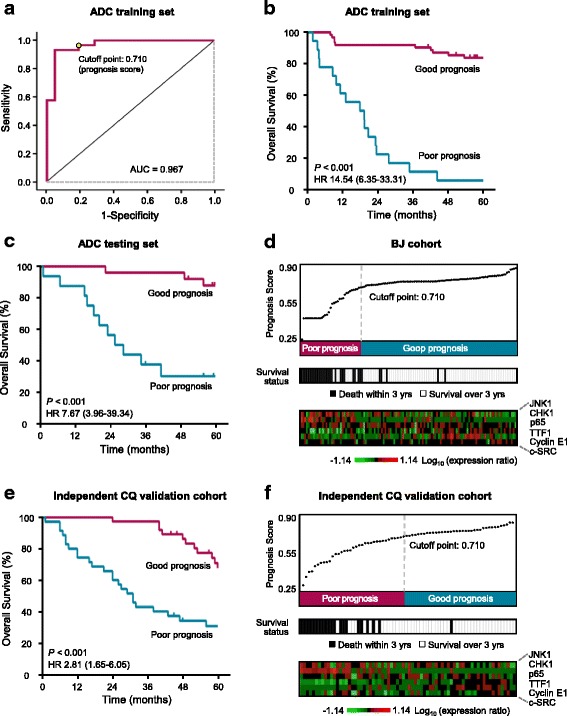
Identification and validation of the 6-protein signature for adeno-carcinoma (ADC). **a** The ROC curve of the ADC training set. The cutoff point of prognosis scores is shown. **b-c** Patients of the training set (**b**) and testing set (**c**) were classified into poor- and good-prognosis groups using the 6-protein ADC signature. The Kaplan-Meier estimates of overall survival for the two predicted prognosis groups are shown. (**d**) The prognosis score distribution, prognosis prediction using the ADC signature, the three-year survival status and the expression profile of the 6-protein signature proteins were summarized on ADC patients of BJ cohort. Each column represents an individual patient. **e** ADC patients of the independent CQ validation cohort were classified into poor- and good-prognosis groups using the 6-protein ADC signature. The Kaplan-Meier survival curves for the two prognosis groups are shown. **f** The prognosis score distribution, prognosis prediction using the signature, the three-year survival status and the expression profile of the 6-protein signature proteins of CQ ADC patients are shown

The performance of the ADC signature in the training set was evaluated by receiver-operating characteristic (ROC) analysis. The value of area under the ROC curve (AUC) of the signature on training set was 0.967 (Fig. 2a), indicating that the signature accurately predicted the prognosis in the training set. Based on the ROC curve, the optimal cutoff point of the prognosis score was calculated as 0.710 to separate good and poor prognosis. A patient with a prognosis score smaller than 0.710 was classified into poor-prognosis group; otherwise, the patient was classified into good-prognosis group. In the training set, the good-prognosis group showed a three-year survival of 91.9% (95% CI, 81.7 to 96.6%) and 11.1% (95% CI, 1.9 to 29.8%) in the poor-prognosis group in Kaplan-Meier analysis (HR 14.54; 95% CI, 6.35 to 33.31; *P* <  0.001, Fig. 2b).

We next evaluated the prognostic performance of this 6-protein signature in the testing set. The result indicated that the ADC signature was strongly associated with overall survival of these patients. Kaplan-Meier analysis showed a three-year overall survival of 96.0% (95% CI, 74.8 to 99.4%) in good-prognosis group, but just 37.5% (95% CI, 15.4 to 59.8%) in poor-prognosis group (HR 7.67; 95% CI, 3.96 to 39.34; *P* <  0.001, Fig. 2c).

The prognosis score distribution, prognosis prediction, three-year survival status and the expression profile of the signature proteins of the ADC patients from BJ cohort are summarized in Fig. 2d. The comparison of the prognosis score distribution (top panel) and the three-year survival data (middle panel) indicated that the 6-protein signature accurately predicted the three-year survival of patients. Particularly, in the distribution of prognosis scores, the top part (32%) and the bottom part (15%) are completely correct in predicting three-year survival, suggesting that the prognosis score is a reliable predictor for prognosis. The expression profile of the six signature proteins was shown in the bottom panel.

### Independent validation of the ADC signature

To further verify the performance of the ADC signature, we used another cohort (CQ cohort) of ADC patients as an independent validation set (Fig. 1, CQ cohort). Kaplan-Meier analysis showed a significant difference in overall survival between the predicted good- and poor-prognosis groups (HR 2.81; 95% CI, 1.65 to 6.05; *P* <  0.001; Fig. 2e). The good-prognosis group had a three-year survival of 97.3% (95% CI, 82.3 to 99.6%), and the poor-prognosis group had a rate of 42.9% (95% CI, 26.4 to 58.3%; Fig. 2e). The prognosis score distribution, prognosis prediction, the survival status, and the expression profile of the six signature proteins of CQ cohort were presented in Fig. 2f. These results further demonstrated the effectiveness of the 6-protein signature in the prognosis prediction of ADC patients.

We compared the prognostic value of ADC signature with that of clinical risk factors, including pathologic stage, age, tumor size, smoking index, and chemotherapy, by both univariate and multivariate Cox regression analysis in independent CQ cohort. The univariate analysis indicated that the 6-protein signature is a better prognostic predictor of three-year overall survival (HR 2.89; 95% CI, 1.52 to 5.48; *P* = 0.001) than the pathologic stage (HR 2.43; 95% CI, 1.64 to 3.60; *P* <  0.001), although both of them are statistically significant (Table 2). The multivariate analysis further showed that the 6-protein signature is an independent predictor (HR 3.07; 95% CI, 1.29 to 7.32; *P* = 0.011) after adjusting for the above risk factors (Table 2).

**Table 2:** Cox regression analysis of overall survival in the independent CQ validation cohort

Histology	Variable	Univariate analysis		Multivariate analysis	
		HR(95% CI)	*P* value	HR(95% CI)	*P* value
ADC	Six-protein signature^a^	2.89 (1.52–5.48)	0.001	3.07 (1.29–7.32)	0.011
	Stage^b^	2.43 (1.64–3.60)	< 0.001	2.43 (1.46–4.03)	0.001
	Age > 60 years	1.33 (0.70–2.53)	0.390	1.37 (0.65–2.87)	0.413
	Tumor size	1.09 (0.91–1.30)	0.341	1.02 (0.84–1.24)	0.880
	Smoking index	1.01 (0.99–1.03)	0.425	0.99 (0.97–1.01)	0.426
	Chemotherapy	1.09 (0.52–2.30)	0.816	1.50 (0.61–3.67)	0.379
SCC	Five-protein signature^a^	10.84 (4.15–28.31)	< 0.001	7.84 (2.88–21.31)	< 0.001
	Stage^b^	2.85 (1.78–4.57)	< 0.001	2.24 (1.26–3.99)	0.006
	Age > 60 years	1.73 (0.86–3.50)	0.127	1.48 (0.70–3.14)	0.307
	Tumor size	1.15 (1.00–1.32)	0.058	1.02 (0.85–1.23)	0.838
	Smoking index	1.00 (0.99–1.01)	0.994	1.00 (0.98–1.02)	0.659
	Chemotherapy	1.23 (0.56–2.71)	0.615	1.45 (0.55–3.83)	0.452

*ADC*, adenocarcinoma; *SCC*, squamous cell carcinoma; *HR*, hazard ratio

^a^Compared with low-risk group. ^b^Modeled as a continuous variable

### Identification and independent validation of the SCC signature

**Fig. 3:**
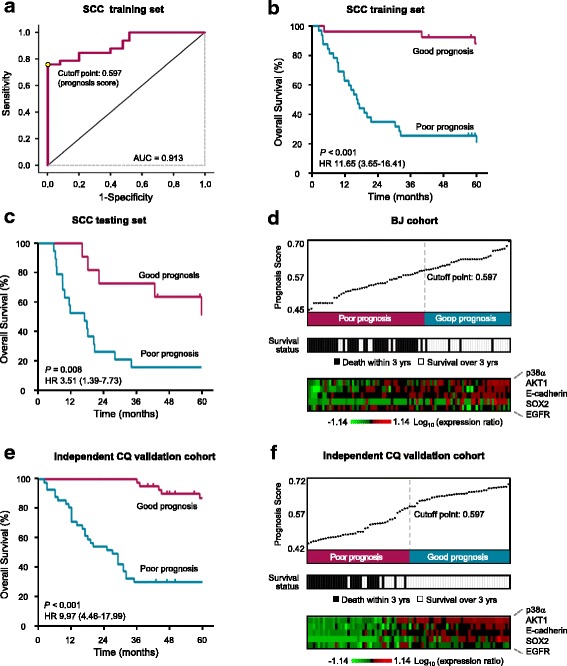
Identification and validation of the 5-protein signature for squamous cell carcinoma (SCC). **a** The ROC curve of the SCC training set. The cutoff point of prognosis scores is shown. **b**-**c** Patients of the training set (**b**) and testing set (**c**) were classified into poor- and good-prognosis groups using the 5-protein SCC signature. The Kaplan-Meier estimates of overall survival for the two predicted prognosis groups are shown. **d** The prognosis score distribution, prognosis prediction using the signature, the three-year survival status and the expression profile of the 5-protein signature proteins were summarized on SCC patients of BJ cohort. Each column represents an individual patient. **e** SCC patients of the independent CQ validation cohort were classified into poor- and good-prognosis groups using the 5-protein SCC signature. The Kaplan-Meier survival curves for the two prognosis groups are shown. **f** Prognosis score distribution, prognosis prediction using the SCC protein signature, patient survival status and the expression profile of the 5-protein signature proteins of CQ SCC patients are shown

Using a similar procedure as in the discovery of the ADC signature, we also identified a protein signature for SCC with BJ cohort (Table 1; Fig. 1; CRD; Additional file 1: Tables S4 and S5). The SCC signature is distinct from the ADC signature and consists of five proteins: EGFR, p38α, AKT1, SOX2, and E-cadherin. An ROC analysis on the training set showed an AUC value of 0.913 and the optimal cutoff point of the prognosis score was 0.597 (Fig. 3a). With this cutoff point, the prognosis prediction of the 5-protein SCC signature in the training set showed a three-year survival of 96.2% (95% CI, 75.7 to 99.5%) in good-prognosis group and 25.0% (95% CI, 11.8 to 40.7%) in poor-prognosis group (HR 11.65; 95% CI, 3.65 to 16.41; *P* < 0.001, Fig. 3b).

The evaluation result of this signature on the testing set indicated that it was strongly associated with overall survival of SCC patients. The three-year overall survival in good-prognosis group was 72.7% (95% CI, 37.1 to 90.3%) and 15.8% (95% CI, 3.9 to 34.9%) in poor-prognosis group (HR 3.51; 95% CI, 1.39 to 7.73; *P* = 0.008; Fig. 3c). The patients with low prognosis scores had significantly more death events than those with high scores, indicating that the SCC signature effectively predicted prognosis (Fig. 3d).

We further used the SCC patients from CQ cohort for independent validation (Fig. 1, CQ cohort). A significant difference in overall survival between the predicted good- and poor-prognosis groups was shown by Kaplan-Meier analysis (HR 9.97; 95% CI, 4.46 to 17.99; *P* < 0.001; Fig. 3e). 97.6% (95% CI, 83.9 to 99.7%) of the patients in good-prognosis group survived at least three years, but only 29.3% (95% CI, 16.4 to 43.4%) of the patients in poor-prognosis group did so (Fig. 3e). Figure 3f showed the prognosis score distribution, prognosis prediction, the survival status, and the expression profile of the five proteins in CQ cohort. Notably, in the distribution of prognosis scores, the top part (50%) and the bottom part (20%) are completely correct in predicting the three-year survival. These results showed that the 5-protein SCC signature accurately predicted the prognosis of patients of CQ cohort. Cox regression analysis further showed that the SCC signature is an independent prognostic factor and has a greater prognostic power than TNM staging system (Table 2).

### Both signatures distinguish between good and poor prognosis within TNM stages

We next investigated whether our signatures could be used to further distinguish between poor- versus good-prognosis groups in each TNM stage (stage I, II, or IIIA). Using the combined samples of the ADC patients from BJ and CQ cohorts, we found that the 6-protein ADC signature could clearly divide the patients into poor- and good-prognosis groups within each stage (Additional file 1: Figure S2 A to C. HR 6.1; 95% CI, 1.81 to 20.27; *P* < 0.0001 for Stage I; HR 3.59; 95% CI, 1.72 to 7.51; *P* = 0.0007 for Stage II; and HR 2.96; 95% CI, 1.43 to 6.11; *P* = 0.015 for Stage IIIA). Similarly, the 5-protein SCC signature also markedly classified SCC patients into good- and poor-prognosis groups within each stage (Additional file 1: Figure S2 D to F. HR 7.36; 95% CI, 2.76 to 19.68; *P* < 0.0001 for Stage I; HR 7.27; 95% CI, 3.28 to 16.08; *P* < 0.0001 for Stage II; and HR 5.13; 95% CI, 2.56 to 10.29; *P* = 0.0020 for Stage IIIA). Taken together, these results indicate that both the ADC and SCC signatures can distinguish between good and poor prognosis within each stage.

### Predicted poor-prognosis ADC patients benefited from adjuvant chemotherapy

**Fig. 4:**
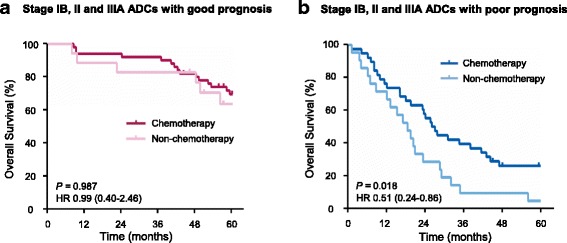
Analysis of adjuvant chemotherapy benefit based on the 6-protein adenocarcinoma (ADC) signature. Kaplan-Meier estimates of overall survival for the patients at stages IB, II and IIIA with or without adjuvant chemotherapy in the good-prognosis (**a**) and poor-prognosis (**b**) group was analyzed

According to the American Society of Clinical Oncology (ASCO) guidelines, adjuvant chemotherapy is recommended for routine use in stage II and IIIA patients [37]. Reports also showed that adjuvant chemotherapy can improve overall survival of NSCLCs in stage IB [38, 39]. However, only a small portion of these patients gain benefit in terms of 5-year survival [40]. To test whether our protein signatures are valuable in selecting patients for adjuvant chemotherapy, we did an exploratory analysis of the predictive value of our prognostic signatures of NLCSCs in stage IB, II and IIIA. For the good-prognosis group classified by the ADC signature, adjuvant chemotherapy did not significantly prolong overall survival of the patients (HR 0.99; 95% CI, 0.40 to 2.46; *P* = 0.987; Fig. 4a). However, for the poor-prognosis group, adjuvant chemotherapy significantly improved survival (HR 0.51; 95% CI, 0.24 to 0.86; *P* = 0.018; Fig. 4b).

We further assessed the capacity of 6-protein ADC and 5-protein SCC signatures to predict benefit from adjuvant therapy when the different stages are analyzed separately. In ADC, we found that adjuvant chemotherapy did not significantly prolong overall survival in either good-prognosis group classified by the ADC signature or poor-prognosis group in stage IB (Additional file 1: Figure S3 A to D). But significant benefit from adjuvant therapy was observed for the poor-prognosis group in stage II and stage IIIA (Additional file 1: Figure S3 E and F. HR 0.36; 95% CI, 0.15 to 0.90; *P* = 0.0134 for Stage II; HR 0.183; 95% CI, 0.02 to 1.56; *P* = 0.0003 for Stage IIIA).

For SCC, we performed similar analysis in all patients or patients within different stages, and did not observe any significant benefit in either good- or poor-prognosis groups. (Additional file 1: Figure S4 and Additional file 1: Figure S5).

### Permutation validation and enrichment analysis

**Fig. 5:**
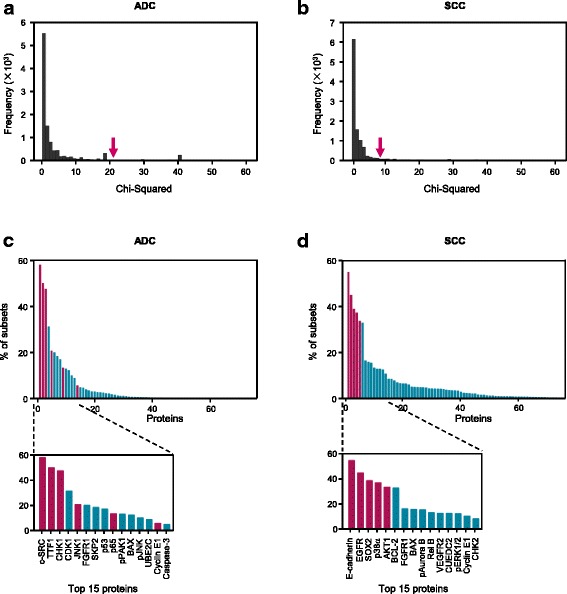
Permutation validation and enrichment analysis. In permutation validation, ten thousand protein combinations were generated randomly. The model with each combination was trained on the training set using SVM. The ability of each combination to separate the testing set into good- and poor-prognosis groups was evaluated using the log-rank test. Histograms of the χ^2^ value from the log-rank test on ADC testing set (**a**) and SCC testing set (**b**) were illustrated. The *x* axis indicates the χ^2^ value. A larger χ^2^ value indicates a lower *P* value and a more statistically significant ability to separate the testing set. The *y* axis shows the frequency and higher values indicate a larger fraction of the population. The performance of the ADC/SCC signature is marked with a red arrow. In enrichment analysis, ten thousand signatures were identified on 10,000 randomly partitioned training sets using random forest algorithm. For each protein, the fraction of the signatures containing the protein (i.e. percentage of subsets) in ADC (**c**) and SCC (**d**) patients from BJ cohort was calculated. A zoom-in on the 15 most enriched proteins is also shown. Each column corresponds to a protein, the signature proteins are denoted in red

The optimal signature proteins were identified using random forest algorithm (RFA) through a large number iterations of signature building and evaluation. RFA is an ensemble learning method composing of thousands of decision trees [33]. In this study, the decision trees were built with patients and proteins selected randomly and independently. RFA can avoid over-fitting and yield small number protein-contained signatures that retain a high predictive accuracy [41]. Permutation analysis showed the efficacy of RFA in our study (Fig. 5a and b). The performances of ADC and SCC signatures were among the top 2.68% and 1.82% in 10,000 randomly generated protein combinations respectively. For each protein, we calculated the percentage of the signatures containing this protein in 10,000 signatures identified using RFA from 10,000 randomly partitioned training sets. The six proteins of the ADC signature are present on the top 15 most highly enriched proteins (Fig. 5c). Especially, the proteins c-SRC, TTF1 and CHK1 are ranked in the top 3. Meanwhile, the five proteins of the SCC signature are among the top five proteins (Fig. 5d). The high frequencies of our signature proteins demonstrated the robustness and the low bias of our signatures.

## Discussion

IHC is currently the most practical method of assessing the expression levels of prognostic and predictive protein biomarkers in tumor cells [42, 43]. Due to the heterogeneity of protein expressions in tumors, the IHC scoring system used in this study considered both the intensity of the staining (richness) and the percentage of positively-stained tumor cells (evenness). We examined the expression levels of 75 signaling proteins representing the most important pathways involved in cancer development (Additional file 1: Table S1). Based on the expression scores of these proteins in lung cancer tissues, we calculated a prognostic score using the SVM algorithm-based model for each patient (Figs. 2 and 3). This score represents the combined information of the expression levels of the signature proteins, 6 proteins for ADC and 5 proteins for SCC. As shown in the distribution of prognosis scores (Fig. 2d and f; Fig. 3d and f), the higher prognostic score indicates more chance of good survival. The results indicated that the prognostic scores might be actionable in the NSCLCs prognosis.

The successful identification of the signatures with excellent performances strongly suggests that NSCLCs at different stages are featured by their specific signaling status, which is represented by expression levels of certain signaling proteins [41, 42]. The good performance of the signatures owed to three important aspects: selection of signaling proteins that play pivotal roles in lung cancer development, reliable and accurate assessment of protein expression levels with IHC staining that distinguishes cancer cells from stromal cells, and the implementation of high-efficient signature identification methods: random forest algorithm and SVM computation.

The prognostic signatures with excellent performance were identified from 75 signaling proteins. The selection of the proteins was based on their known importance in cancer development and prognosis prediction, and availability and suitability of a corresponding antibody for paraffin-embedded tissues. Although these proteins show some prognostic values, however, none of them individually predicts accurately in clinical practice [5, 43], as CQ Zhu et al. summarized. Using a signature of multiple proteins will likely overcome the limitation of single protein as prognostic predictors because the multiple-protein signature may reflect the heterogeneity of tumourigenesis. In this study, we identified the signatures with multiple proteins which have effective prognostic values in NSCLCs. Both 6-protein ADC signature and 5-protein SCC signature performed much better than each of the signature protein (Additional file 1: Figure S6 and Additional file 1: Figure S7). We noticed that the ADC signature does not include some top ranked proteins. One possible reason is that these proteins have functional redundancies with some ADC signature proteins. For example, CDK1 correlates with CHK1 in regulating G2/M transition and SKP2 is also a key regulator of cell cycle [44, 45]. The inclusion of these redundant proteins in the signature will limit its ability to reflect the contribution of multiple important pathways to the complexity of cancer.

Over-treating with adjuvant chemotherapy of cancer patients is a major concern. Only a survival advantage of 5.4% was found in Lung Adjuvant Cisplatin Evaluation meta-analysis [40]. Therefore, it is necessary to develop more accurate tool for identifying patients most likely to benefit from adjuvant chemotherapy. The ADC prognostic signature in this study can identify a poor-prognosis subset from stage II-IIIA patients who could benefit from adjuvant chemotherapy. Meantime, the ADC signature also showed that the good-prognosis patients do not benefit from adjuvant chemotherapy. Hence, using ADC signature to identify patients for the treatment may spare the patients with good prognosis from adjuvant chemotherapy and avoid over-treatment of lung cancer patients.

The signatures identified in the study not only provide a tool for better prognosis prediction, but also help to reveal novel roles of the signature proteins in the development of lung cancer (Additional file 1: Table S6). For example, the upregulation of JNK1 and CHK1 are known to promote breast cancer metastasis [46]. Our study suggests that they might play similar roles in lung cancer metastasis, as the majority of ADC patients with poor prognosis had high JNK1 and CHK1 expression levels, whereas most ADC patients with good prognosis had low CHK1 expression. As we have identified several patients with good prognosis but high JNK1 expression, low CHK1 expression may have a dominant effect on tumor progression, possibly by promoting metastasis (Fig. 2d and f). Further studies will help to determine how these signature proteins cooperate to regulate NSCLC progression.

## Conclusions

To our knowledge, this is one of the best identification of protein signatures that precisely predict the prognosis of NSCLC patients. Both signatures contain only a small number of proteins, six for ADC and five for SCC. This makes the application of the signatures more practical in routine clinical application. However, a prospective study with larger sample size cohorts from multiple centers, especially including non-Asian cohorts, will be needed to further validate the performance of the signatures. Nevertheless, our study demonstrated that signaling protein signatures are obviously valuable in prognosis prediction of lung cancer.

## Additional file

Additional file 1: Supplementary Figures, Tables and Methods. (PDF 4956 kb)

## Abbreviations

ADC: Adenocarcinoma
ASCO: American Society of Clinical Oncology
AUC: Area under the curve
BJ: Beijing
CI: Confidence interval
CQ: Chongqing
CRD: Clinical research database
H&E: Hematoxylin and eosin
HR: Hazard ratio
IHC: Immunohistochemistry
NSCLC: Non-small-cell lung cancer
OS: Overall survival
RBF: Radial basis function
RFA: Random forest algorithm
ROC: Receiver-operating characteristic
SCC: Squamous cell carcinoma
SVM: Support vector machines
TNM: Tumor-node-metastasis
