# Supplementary material for: Signaling protein signature predicts clinical outcome of non-small-cell lung cancer
Source: BMC Cancer. 2018 Mar 6;18:259. doi: 10.1186/s12885-018-4104-4 (PMC5840771; doi:10.1186/s12885-018-4104-4)
Supplement: Supplementary file 1 — Supplementary Figures, Tables and Methods. (PDF 4956 kb) [file 12885_2018_4104_MOESM1_ESM.pdf]

Signaling protein signature predicts clinical outcome of non-small-cell lung cancer

Bao-Feng Jin, Fan Yang, Xiao-Min Ying, Lin Gong, Shuo-Feng Hu, Qing Zhao, Yan-Hong Tai, Yi-Da Liao, Ke-Zhong Chen, Yuan Cao, Teng Li, Xiao Li, Yan-Huang, Xiao-Yan Zhan, Xuan-He Qin, Jin Wu, Shuai Chen, Sai-Sai Guo, Yu-Cheng Zhang, Jing Chen, Dan-Hua Shen, Kun-Kun Sun, Lu Chen, Wei-Hua Li, Ai-Ling Li, Na Wang, Qing Xia, Jun Wang, Tao Zhou

Seven additional figures and six additional tables

Figure S1. Schematic diagram of IHC marker selection.

Figure S2. Prognosis classification according to the 6-protein ADC and 5-protein SCC signatures within each stage (stage I, II or IIIA)

Figure S3. Analysis of adjuvant chemotherapy benefit to patients within different stages based on 6-protein ADC signature.

Figure S4. Analysis of adjuvant chemotherapy benefit based on the 5-protein squamous cell carcinoma (SCC) signature.

Figure S5. Analysis of adjuvant chemotherapy benefit to patients within different stages based on 5-protein SCC signature.

Figure S6. ROC curves of the prognostic accuracy of the ADC signature and each protein in the signature in the ADC training set.

Figure S7. ROC curves of the prognostic accuracy of the SCC signature and each protein in the signature in the SCC training set.

Table S1. Proteins tested via immunohistochemistry on tissue microarrays in this study.

Table S2. Scaling coefficients of the ADC model.

Table S3. Support vectors and related coefficients of the ADC model.

Table S4. Scaling coefficients of the SCC model.

Table S5. Support vectors and related coefficients of the SCC model.

Table S6. Signature proteins and their related pathways or functions in tumor progression.

Additional methods

Data process of missing values

The ADC prognostic model

The SCC prognostic model

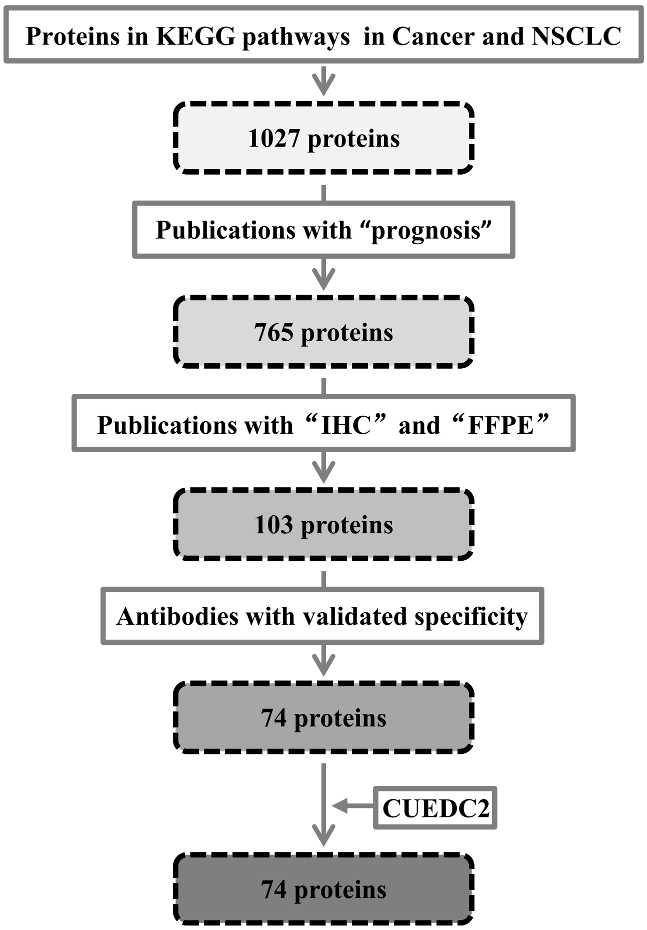

Figure S1. Schematic diagram of IHC marker selection.

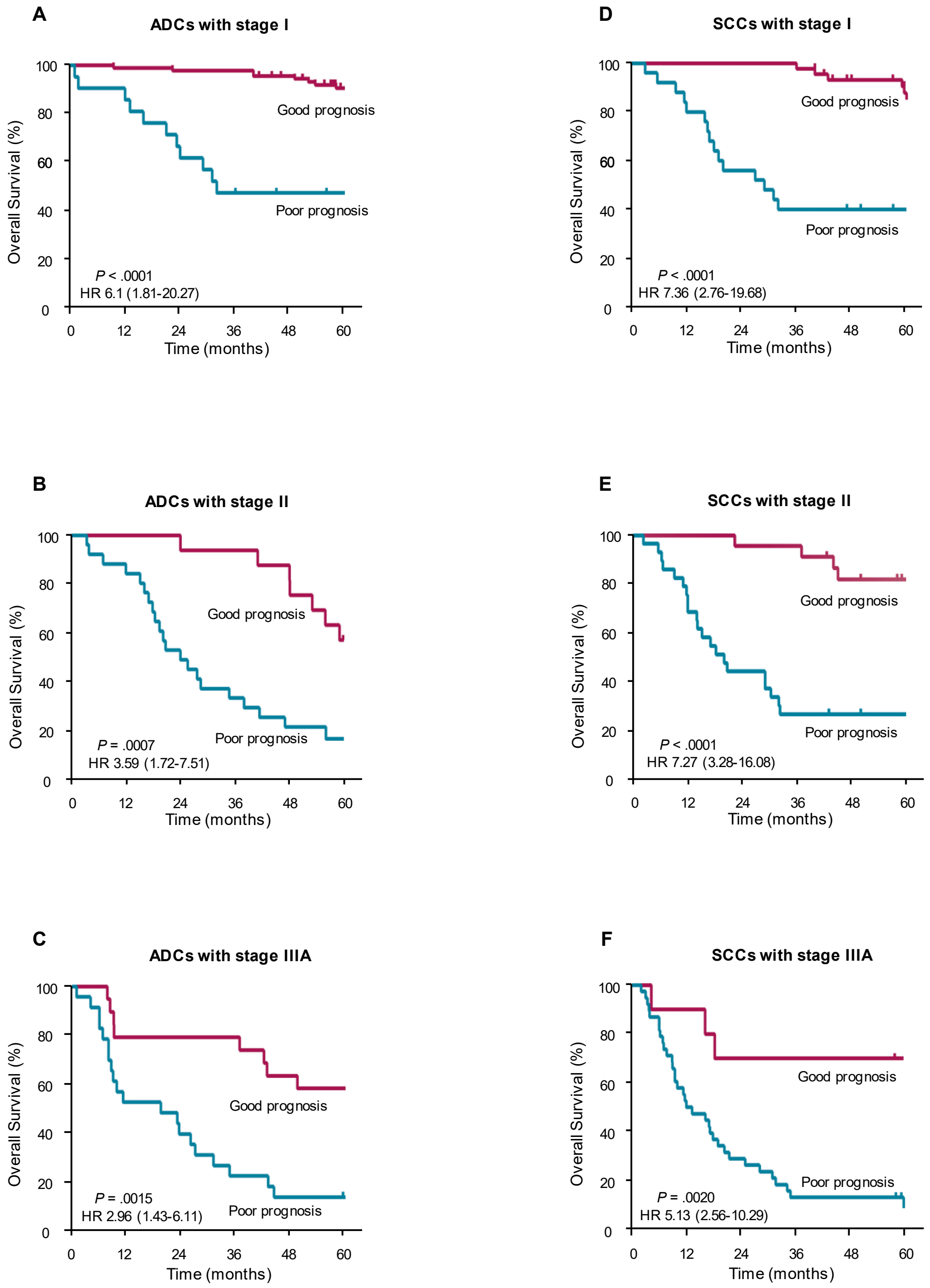

Fig S2. Prognosis classification according to the 6-protein ADC and 5-protein SCC signatures within each stage (stage I, II or IIIA).

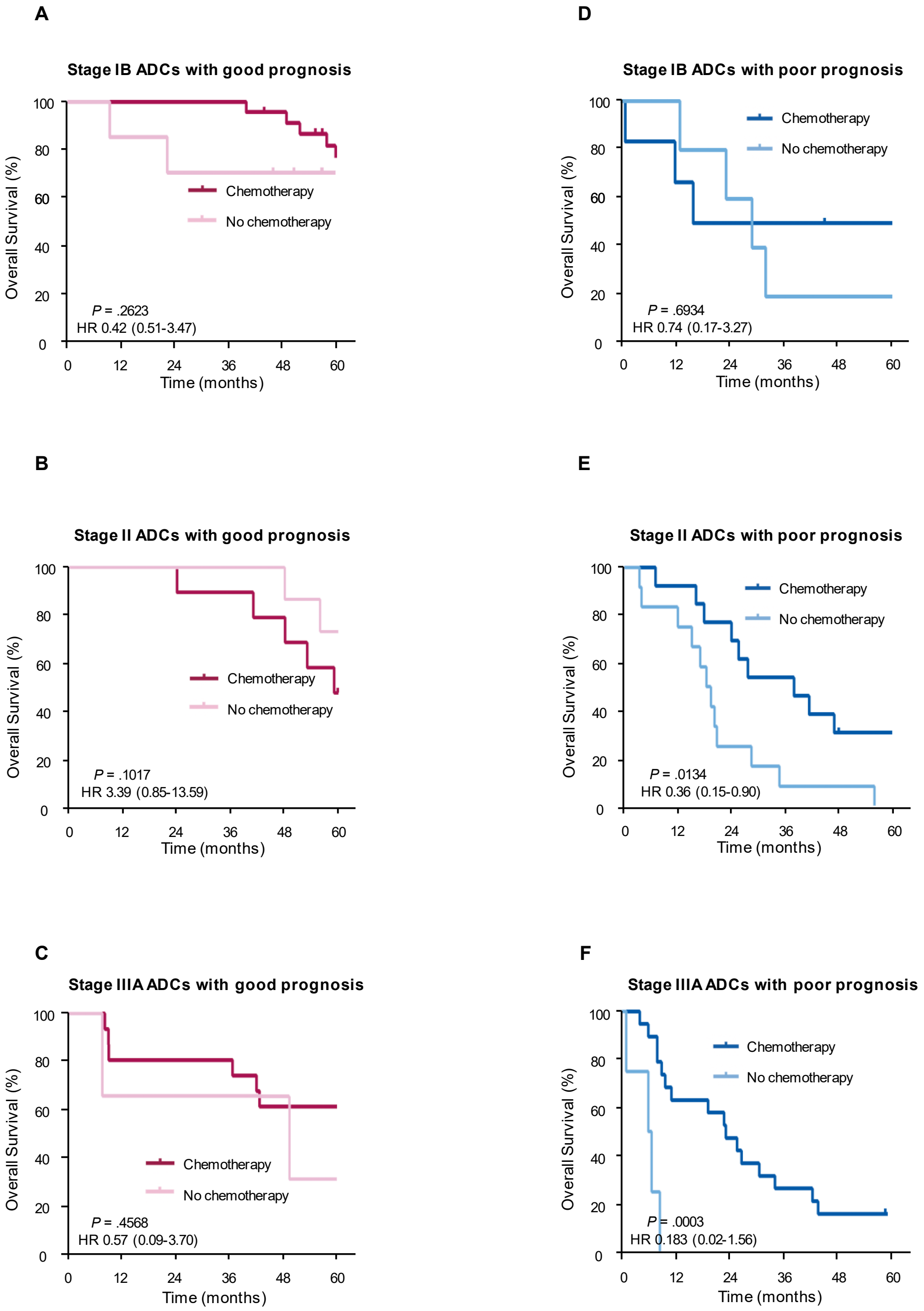

Fig S3. Analysis of adjuvant chemotherapy benefit to patients within different stages based on 6-protein ADC signature.

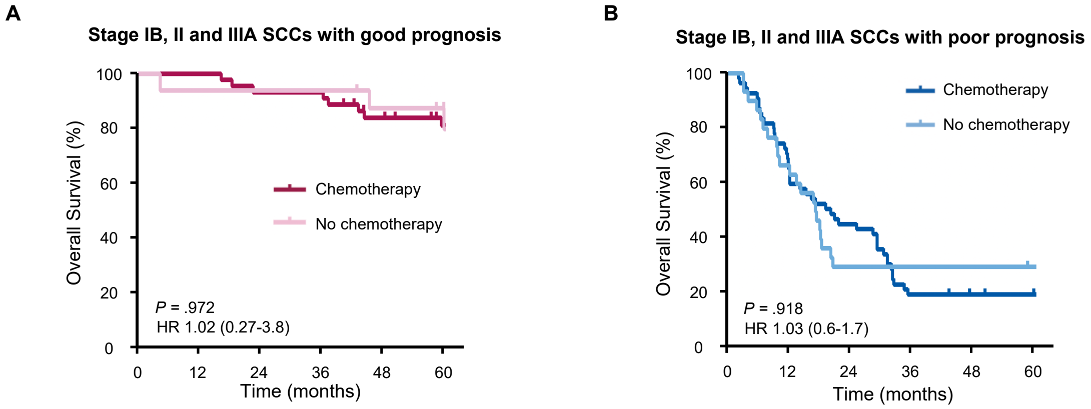

Figure S4. Analysis of adjuvant chemotherapy benefit based on the 5-protein squamous cell carcinoma (SCC) signature. The overall survival of the patients with or without adjuvant chemotherapy in the good-prognosis (**A**) and poor-prognosis (**B**) group was analyzed.

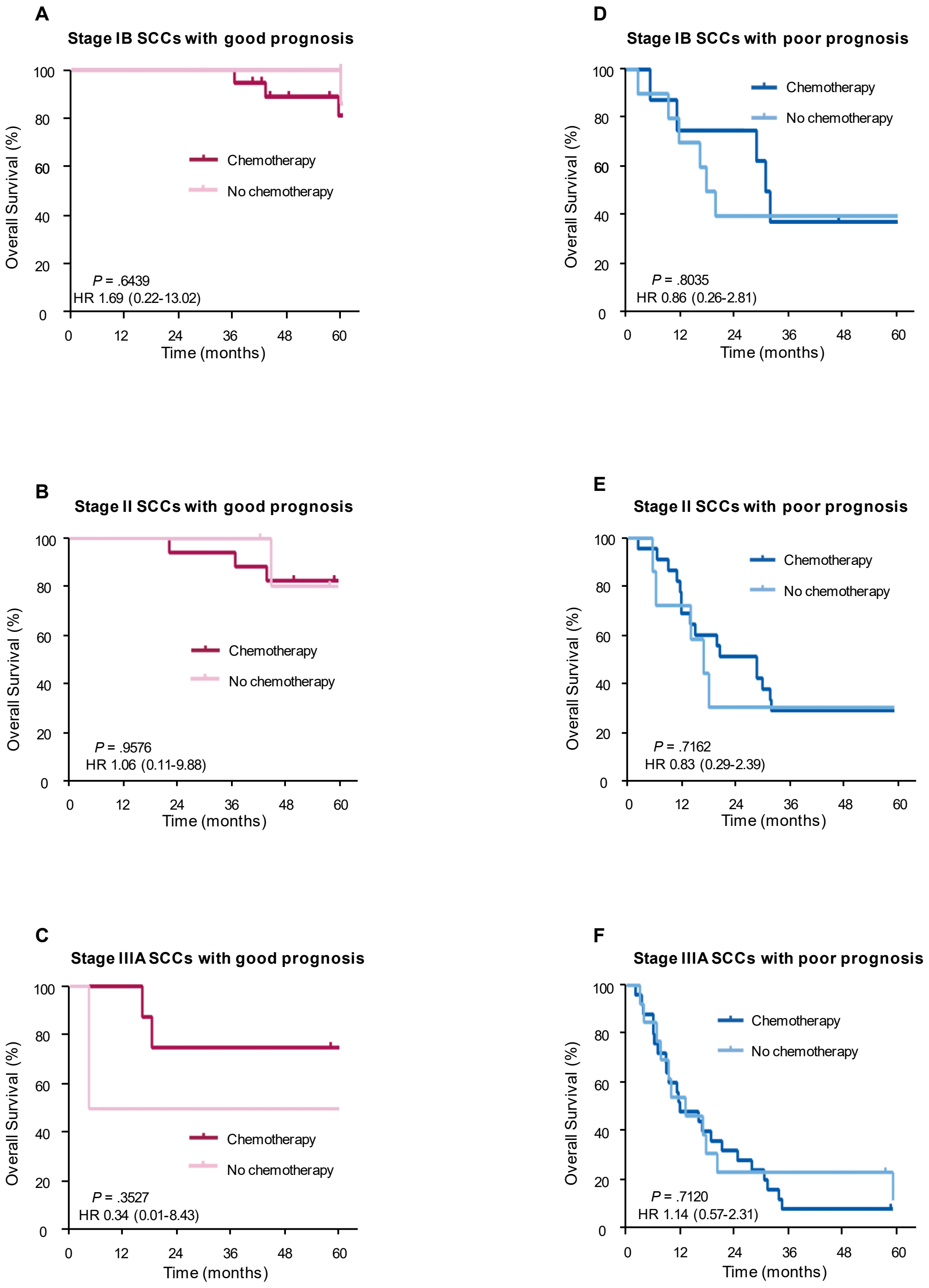

Fig S5. Analysis of adjuvant chemotherapy benefit to patients within different stages based on 5-protein SCC signature.

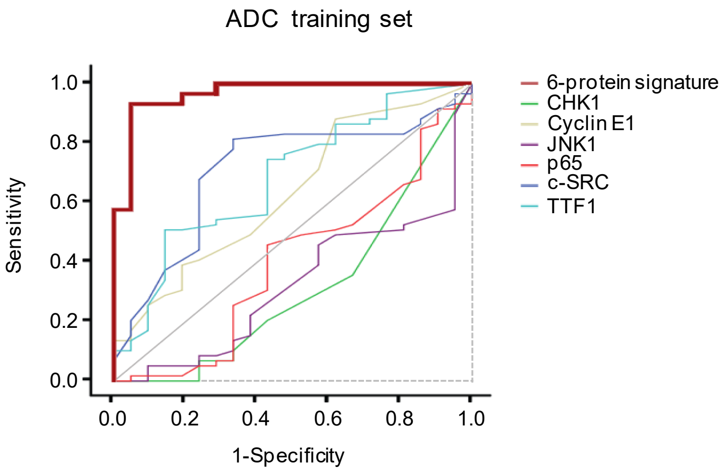

Figure S6. ROC curves of the prognostic accuracy of the ADC signature and each protein in the signature in the ADC training set.

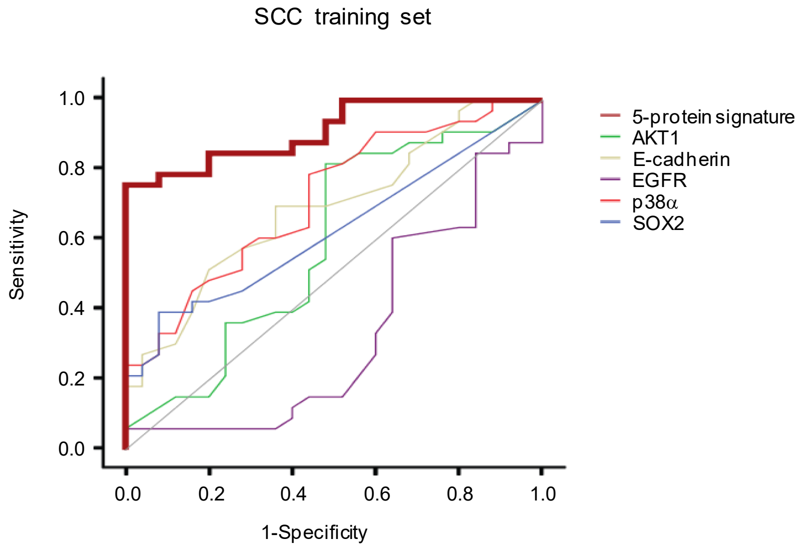

Figure S7. ROC curves of the prognostic accuracy of the SCC signature and each protein in the signature in the SCC training set.

Table S1. Proteins tested via immunohistochemistry on tissue microarrays in this study.

Protein	Ab	Provider	Cat. #	Retrieval	Dilution	Localization	References
AKT1	Rmab	Epitomics	#1085-1	Citrate	1/2500	whole cell*	[1]
pAKT1 (S473)	Rmab	CST	#4060	Citrate	1/50	whole cell	[2]
pAMPKa (T172)	Rmab	CST	#2535	Citrate	1/50	whole cell	[3]
pAurora B (T232)	Rpab	Rockland	600-401-677S	Citrate	1/200	nucleus	[4]
ALK	Rmab	CST	#3633	EDTA	1/100	cell membrane, cytoplasm	[5,6]
Aurora A	Rmab	Epitomics	#1800-1	Citrate	1/50	whole cell	[7]
BAX	Rmab	CST	#5023	Citrate	1/200	whole cell	[8]
BCL-2	Rmab	Epitomics	#1017-1	Citrate	1/200	cytoplasm	[9–12]
BCL-xl/BCL2L1	Rmab	CST	#2764	Citrate	1/100	mitochondria	[13,14]
β-catenin	Rmab	CST	#8480	Citrate	1/150	mainly cytoplasm	[15]
Caspase-3	Rmab	Epitomics	#1475-1	Citrate	1/200	cytoplasm	[16]
Caspase-8	Rmab	Epitomics	#3614-1	Citrate	1/300	cytoplasm	[17]
CDH1/FZR1	Mmab	Abnova	H00051343-M02	Citrate	1/400	nucleus	[18]
CDK1/CDC2	Rmab	Epitomics	#3787-1	Citrate	1/100	whole cell	[19,20]
CHK1/CHEK1	Mmab	CST	#2360	Citrate	1/100	nucleus	[21]
CHK2	Mmab	CST	#3440	Citrate	1/2000	nucleus	[22]
c-Kit/CD117	Rmab	Epitomics	#1522-1	Citrate	1/100	cell membrane, cytoplasm	[23,24]
c-MET	Rmab	Epitomics	#1996-1	Citrate	1/400	cell membrane	[25–27]
c-MYC	Rmab	Epitomics	#1472-1	Citrate	1/100	nucleus	[28–30]
COX2	Rpab	CST	#4842	Citrate	1/200	whole cell	[31]
c-SRC	Rmab	CST	#2109	EDTA	1/1000	mainly cytoplasm	[32]
CUEDC2	Mmab	Homemade	c5-19	NO	1/800	whole cell	[33]
CXCR4	Rmab	Epitomics	#3108-1	EDTA	1/100	cell membrane, cytoplasm	[34]
Cyclin D1/CCND1	Rmab	Epitomics	#2261-1	Citrate	1/150	nucleus	[35,36]
Cyclin E1/CCNE1	Rpab	Abnova	PAB4852	Citrate	1/100	nucleus	[37]
DCLK1	Rmab	Epitomics	#3842-1	Citrate	1/100	cytoplasm	[38]
DDR1	Rmab	CST	#5583	Citrate	1/1600	cell membrane, cytoplasm	[39–41]
DNA-PK/PRKDC	Rmab	Epitomics	#1579-1	Citrate	1/100	nucleus	[42,43]
E-cadherin/CDH1	Mmab	BD	610182	Citrate	1/300	cell junction	[44–46]
pERK1/2(T202/T204)	Rmab	CST	#4376	Citrate	1/200	whole cell	[47]
EGFR	Rmab	Epitomics	#1902-1	NO	1/100	cell membrane	[48]
EGFR (∆E746-A750)	Rmab	CST	#2085	EDTA	1/100	cell membrane	[49]
EGFR (L858R)	Rmab	CST	#3197	EDTA	1/100	cell membrane	[50]
ErbB2/HER2	Rmab	Epitomics	#2064-1	NO	1/50	cell membrane	[51]
FGFR1	Rmab	CST	#9740	EDTA	1/150	cell membrane, cytoplasm	[52,53]
pFOXO3A (S253)	Rpab	Abnova	PAB16950	Citrate	1/100	mainly nucleus	[54]
pGSK-3β (S9)	Rmab	Epitomics	#2435-1	Citrate	1/100	whole cell	[55]
HDAC2	Rpab	Epitomics	#S2398	Citrate	1/100	nucleus	[56,57]
HDAC3	Rpab	Abcam	ab16047	Citrate	1/100	nucleus	[58,59]
HIF-1α	Rmab	Epitomics	#2015-1	Citrate	1/200	nucleus	[60–62]
HSP90	Rpab	CST	#4875	EDTA	1/50	cytoplasm	[63–65]
JNK/JNK1/MAPK8	Mmab	BD	51-1570GR	Citrate	1/100	whole cell	[66]
pJNK (T183/T185)	Mmab	Santa Cruz	sc-6254	Citrate	1/200	whole cell	[67,68]
pJAK2 (Y1007)	Rmab	Epitomics	#1477-1	Citrate	1/150	mainly nucleus	[69,70]
LKB1/STK11	Rpab	Proteintech	10746-1-AP	Citrate	1/100	whole cell	[71]
LSD1/AOF2	Mmab	CST	#4064	Citrate	1/100	nucleus	[72–74]
MAD2	Rpab	Epitomics	#S0224	EDTA	1/300	nucleus	[75–77]
MDM2	Rpab	Epitomics	#S1357	NO	1/200	whole cell	[78–80]
mTOR	Rmab	Epitomics	#1612-1	Citrate	1/200	mainly cytoplasm	[81,82]
pmTOR (S2448)	Rmab	Epitomics	#2936-1	Citrate	1/100	whole cell	[83]
NF-κB/p65	Rmab	Epitomics	#1546-1	Citrate	1/400	cytoplasm, nucleus	[84,85]
p16/INK4a	Rmab	Epitomics	#3562-1	Citrate	1/200	whole cell	[86,87]
p300/KAT3B	Mmab	Abcam	ab54984	Citrate	1/3000	nucleus	[88]

(continued on next page)

Protein	Ab	Provider	Cat. #	Retrieval	Dilution	Localization	References
p38α/MAPK14	Rpab	Santa Cruz	sc-535	Citrate	1/100	whole cell	[89]
p53	Mmab	Santa Cruz	sc-126	Citrate	1/150	nucleus	[90–93]
p53BP1	Mmab	BD	612523	Citrate	1/100	nucleus	[94]
PAK1	Rpab	CST	#2602	EDTA	1/150	mainly cytoplasm	[95]
PDGFRa	Rpab	CST	#3164	EDTA	1/100	cell membrane, cytoplasm	[96]
PI3K p110α	Rmab	CST	#4249	Citrate	1/400	mainly cytoplasm	[97,98]
PLK1	Rmab	Epitomics	#6696-1	Citrate	1/200	mainly cytoplasm	[99,100]
PPARγ/PPARG	Rpab	Santa Cruz	sc-7196	Citrate	1/100	whole cell	[101–103]
PTEN	Rmab	CST	#9188	Citrate	1/100	mainly cytoplasm	[104–106]
pp21 (S146)	Rmab	Epitomics	#2674-1	Citrate	1/400	whole cell	[107,108]
pPAK1 (S144)	Rmab	Epitomics	#1705-1	Citrate	1/1000	mainly cytoplasm	[109]
Raf1/c-Raf	Rpab	Santa Cruz	sc-133	Citrate	1/200	cell membrane, cytoplasm	[110,111]
Rel B	Mmab	Santa Cruz	sc-48366	Citrate	1/100	nucleus	[112]
SKP2	Rpab	Santa Cruz	sc-7164	Citrate	1/100	nucleus	[113,114]
pSTAT3 (Y705)	Rmab	Epitomics	#2236-1	EDTA	1/100	mainly nucleus	[115,116]
SLC2A1/GLUT1	Rpab	Abnova	PAB12031	Citrate	1/800	mainly cytoplasm	[117,118]
SMAD2/MADH2	Rmab	CST	#3122	Citrate	1/100	whole cell	[119]
SOX2	Rmab	Epitomics	#2696-1	Citrate	1/50	nucleus	[120–122]
Survivin	Rmab	CST	#2808	Citrate	1/400	nucleus	[123,124]
TTF1/NKX2.1	Rmab	Epitomics	#5883-1	Citrate	1/500	nucleus	[125]
UBE2C/UBCH10	Mmab	Abcam	ab56861	Citrate	1/150	whole cell	[126]
VEGFR2	Rmab	CST	#9698	Citrate	1/1000	cell membrane	[127]

* “whole cell” in localization column (column 7) denotes cell membrane, cytoplasm and nucleus.

Table S2. Scaling coefficients of the ADC model.

Protein	Lower	Upper
CHK1	-0.166273865	1.037846118
Cyclin E1	-0.498933141	0.823286154
JNK1	-0.694643566	0.584110035
p65	-1.246927602	0.237372238
c-SRC	-0.917718271	0.161462975
TTF1	-1.106011396	0.236411285

Table S3. Support vectors and related coefficients of the ADC model.

No.	SV coefficient	SV					
		CHK1	Cyclin E1	JNK1	p65	c-SRC	TTF1
1	1.166529102994991	-1	-0.089319	-0.529182	-1	0.722452	-1
2	8	-1	-0.398073	0.0216394	0.980492	0.83288	0.773408
3	7.914210883170862	-1	-0.54466	0.321753	0.640482	0.580274	0.722452
4	6.420936990446873	-1	0.177038	0.492457	0.859747	0.722452	0.0413561
5	5.721178217158177	-1	-1	-0.529182	0.821980	0.722452	0.521412
6	6.751657750534642	-0.707519	-0.0119453	0.217046	0.723039	0.536913	-0.551512
7	8	0.0849625	0.177038	0.321753	0.781607	0.566184	0.851932
8	1.358361658604497	0.229716	0.443395	0.742233	0.80883	0.832880	0.634224
9	0.3683819003162858	-0.5	-1	-1	0.738240	0.506537	0.557368
10	8	-1	-0.544660	0.861706	0.821980	0.594427	0.777569
11	1.531517270194687	-1	0.934253	-0.377613	0.781607	0.768456	0.985125
12	1.367992469618161	-1	-0.398073	-0.724589	0.795376	0.983055	-0.737652
13	4.168716626409085	-0.0465547	-0.398073	0.217046	0.883636	0.853257	-0.830242
14	0.05178166562459967	-0.339036	0.323625	-0.377613	0.723039	1	0.813861
15	6.027605190470098	-0.5	-0.733643	-0.253771	0.252333	0.83288	0.712157
16	8	-0.707519	-0.398073	0.321753	0.478017	0.853257	0.384689
17	6.911852250434124	-0.707519	-0.278302	-0.253771	0.883636	-0.262515	0.489844
18	1.247882046206535	-1	-0.278302	-0.724589	0.544335	0.594427	0.384689
19	3.850457244809196	-1	-0.544660	0.321753	0.622475	0.407860	0.870160
20	8	-0.5	-0.54466	0.687864	0.883636	0.722452	-1
21	7.766849090380986	-1	-0.733643	0.816406	0.917734	0.873131	0.756491
22	6.980436885497707	-0.5	-0.398073	-0.529182	0.723039	0.768456	0.938331
23	0.619650775859574	-0.5	-0.398073	-0.724589	0.960311	0.698425	0.938331
24	0.1309579496779729	-1	-0.398073	0.217046	0.871813	0.698425	1
25	-0.5460730718184561	0.850220	-0.3980730	0.767868	0.928672	0.722452	-1
26	-8	-0.707519	-0.278302	0.0932051	0.723039	0.832880	0.851932
27	-0.8807924966875877	0.543731	0.0572679	0.0216394	0.738240	0.929968	0.730257
28	-2.7642169646283490	0.584962	-0.398073	0.839434	0.723039	0.853257	-1
29	-8	-1	-0.733643	0.0216394	0.767505	0.83288	0.905143
30	-6.456400904965796	-0.707519	0.177038	1	0.707434	0.873131	-1
31	-8	-0.707519	-0.278302	0.981886	0.285781	0.621713	0.259064
32	-8	-0.5	-0.733643	0.0216394	0.767505	0.621713	0.449808
33	-0.8397050269053298	-1	-1	0.321753	0.970489	0.673658	-1
34	-8	-0.707519	-1	0.839434	0.970489	0.673658	0.793951
35	-8	-1	-0.398073	-0.253771	0.960311	0.722452	0.489844
36	-8	-0.707519	-0.733643	0.321753	0.753055	0.506537	-1.000000
37	-8	-1	-0.177038	0.0216394	0.821980	0.594427	-0.0268154
38	-2.325671665357503	-0.207519	-0.398073	0.742233	0.970489	0.621713	0.489844
39	-7.282395004410535	0.292481	-0.0119453	-0.253771	0.478017	0.648104	0.707552
40	-8	-1	-0.54466	0.321753	0.723039	0.621713	0.707552
41	-5.057743931568415	-1	-0.278302	-1	0.216857	-0.185804	0.159324
42	-8	-1	0.278302	0.492457	0.949954	0.536913	0.707552
43	-4.203956902067077	-0.207519	-0.733643	-0.253771	0.753055	0.648104	0.734704

Table S4. Scaling coefficients of the SCC model.

Protein	Lower	Upper
AKT1	-1.131249036	0.211173644
E-cadherin	-0.791163384	0.178873393
EGFR	-1.212120146	0.250277851
p38α	-0.970134933	0.352084361
SOX2	-0.650762327	0.639272285

Table S5. Support vectors and related coefficients of the SCC model.

No.	SV coefficient	SV				
		AKT1	E-cadherin	EGFR	p38α	SOX2
1	0.8464156345297407	-1	0.664493	-1	0.405847	-1
2	0.2124651100093301	-0.0268154	0.57915	-1	0.709752	-1
3	0.7168128272667304	0.421672	0.823862	-0.588306	0.278302	0.927139
4	1.185442908741702	-1	0.843119	0.545863	0.478913	0.823348
5	2	0.707552	0.898523	0.733005	0.98417	-1
6	2	-1	0.664493	0.627918	0.733643	-1
7	1.664321410011921	0.752192	0.916255	0.682783	0.951317	-1
8	0.1954537223007999	0.730257	0.898523	0.682783	0.512608	0.982511
9	2	0.730257	0.664493	0.567464	0.778966	-1
10	2	0.730257	0.49891	0.733005	0.366021	-1
11	2	0.259064	0.441121	0.567464	-0.278302	-1
12	2	0.659602	0.49891	0.567464	1	-1
13	2	0.580274	0.823862	0.808289	0.278302	-1
14	2	0.607811	0.698717	0.733005	0.68496	-1
15	2	0.954308	0.950698	0.608442	0.443395	0.206404
16	2	0.634224	0.553194	0.98967	0.733643	0.0836453
17	2	0.345463	0.470481	1	-0.398073	-1
18	2	0.684021	0.950698	0.545863	0.478913	-1
19	2	0.833177	0.57915	0.682783	0.861187	-1
20	2	0.833177	0.861971	0.7	0.632379	-1
21	2	0.634224	-0.121747	0.523448	0.632379	0.0836453
22	2	0.793951	0.843119	0.665051	0.478913	-1
23	2	0.887888	0.553194	0.7	-0.0119453	-0.533299
24	-2	0.887888	0.604375	0.8876	0.709752	-1
25	-0.7048208520469	-1	-1	-0.455771	-0.0119453	-1
26	-2	0.92195	0.861971	0.911846	0.861187	-1
27	-2	0.793951	0.933642	0.911846	0.733643	-1
28	-2	0.954308	0.676046	0.83592	-0.089319	-1
29	-2	0.905143	0.843119	0.83592	0.323625	0.0127054
30	-2	0.793951	0.57915	0.608442	0.54466	-1
31	-2	0.752192	0.57915	0.588306	0.756695	-1
32	-2	0.707552	0.652788	0.979158	0.575219	-0.533299
33	-1.196660866524577	0.303705	-0.542597	-0.455771	-0.0119453	-1
34	-2	0.303705	0.823862	0.608442	0.512608	-1
35	-0.6884342289129464	0.0413561	0.49891	0.733005	0.366021	-0.533299
36	-2	-0.103025	0.698717	0.764273	0.68496	-1
37	-1.295112591246491	0.938331	-0.542597	0.733005	0.68496	-1
38	-0.9318297032828035	0.345463	0.57915	0.8876	0.0572679	-1
39	-0.6982911187307125	-1	-0.861971	0.862322	-0.54466	-1
40	-2	0.384689	0.57915	0.748845	0.22962	-1
41	-2	0.813861	0.470481	0.911846	0.88023	-1
42	-0.8260630063038303	-0.103025	0.676046	0.0642127	-1	0.356648

(continued on following page)

No.	SV coefficient	SV				
		AKT1	E-cadherin	EGFR	p38α	SOX2
43	-2	0.259064	0.652788	0.748845	0.512608	-0.383055
44	-1.473497015010048	-0.103025	0.861971	0.808289	0.366021	-1
45	-1.006202230801914	-1	0.553194	0.7	-0.54466	-0.156506
46	-2	0.9699	0.698717	0.450628	-0.278302	0.51581
47	-2	0.773408	0.49891	0.608442	0.278302	-1

Table S6. Signature proteins and their related pathways or functions in tumor progression.

Histology	Proteins in signature	Aliases	Reference sequence	Protein location	Relevant biological functions and pathways
**ADC**					
	c-SRC	SRC1	NP_005408.1	cytoplasm	Gene transcription, immune response, cell adhesion, cell cycle progression, apoptosis, migration, and transformation
	Cyclin E1	CCNE1	NP_001229.1	nucleus	Essential for the control of the cell cycle at the G1/S (start) transition
	TTF1	NKX2.1	NP_001192225.1	nucleus	Regulation of thyroid specific genes, maintenance of the thyroid differentiation phenotype, lung development and surfactant homeostasis
	p65	Rel A	NP_001158884.1	cytoplasm	NF-κB pathway, involved in any biological processes such as inflammation, immunity, differentiation, cell growth, tumorigenesis and apoptosis
	CHK1	CHEK1	NP_001107593.1	nucleus	Checkpoint-mediated cell cycle arrest, S to G2/M phase transition and DNA repair.
	JNK1	MAPK8	NP_001265476.1	whole cell	MAPK pathway, involved in various processes such as cell proliferation, differentiation, migration, transformation and programmed cell death
**SCC**					
	EGFR	ERBB1	NP_005219.2	cell membrane	Gene expression, cytoskeletal rearrangement, anti-apoptosis and increased cell proliferation
	SOX2	ANOP3	NP_003097.1	nucleus	Embryonic development, embryonic stem cell pluripotency, as a switch in neuronal development.
	E-cadherin	CDH1	NP_004351.1	cell junction	Regulating cell-cell adhesions, mobility and proliferation of epithelial cells
	AKT1	PKBα	NP_001014431.1	whole cell	Regulating many processes including metabolism, proliferation, cell survival, growth and angiogenesis
	p38α	MAPK14	NP_001306.1	whole cell	Involved in a wide variety of cellular processes such as proliferation, differentiation, transcription regulation and development

Methods

Data process of missing values

After IHC staining and evaluation, protein expression profiles were obtained. In 211 tissue samples of BJ cohort, 75 proteins/phospho-proteins were detected. Two samples were removed because more than 1/3 proteins were not successfully detected, which resulted in 209 samples in BJ cohort. In 173 tissue samples of CQ cohort,19 samples with more than 1 missing value were removed and 154 samples were left for further analysis. Among 15,675 expression values (209 × 75) of BJ cohort, there were 418 missing values. The missing value rate was 2.7% (418/15,675). Each sample has 2 missing values in average (418/209) in all 75 expression values. In 154 tissue samples of CQ cohort, 6 proteins for 72 ADC samples and 5 proteins for 82 SCC samples were detected. Among 842 expression values (72 × 6 + 82 × 5), there were 21 missing values. The missing value rate was 2.5% (21/842). Missing values of BJ dataset were replaced with the median score of the respective protein in all tumors. For independent validation of CQ dataset, missing values were replaced with the median score of the corresponding protein in BJ dataset.

The ADC prognostic model

Clinical implementation of the 6-protein ADC signature would be straightforward. For each ADC patient, IHC would be performed for the 6 signature proteins. After normalization, the expression levels of the 6 proteins would be scaled as:

$$f_s(x) = -1 + 2 \cdot (x - lower) / (upper - lower), \tag{1}$$

where x is the normalized expression level, and upper and lower for each protein are listed in Table S2.
The probability of each patient would be calculated as:

$$prob(x) = \frac{1}{1 + \exp(-0.669914 \cdot f(x) - 0.468554)}, \tag{2}$$

where $f(x)$ is calculated as:

$$f(x) = \sum_{i=1}^{43} sv\_coef(i) \cdot \exp(-0.5 \cdot \|SVs(i) - x\|^2) - 0.0192488. \tag{3}$$

In this equation, $SVs(i)$ and $sv\_coef(i)$ are support vectors and related coefficients (Table S3).

When the probability calculated is smaller than the preset threshold of 0.710, the patient is classified into poor-prognosis group; otherwise, the patient is classified into good-prognosis group.

The SCC prognostic model

For each SCC patient, IHC would be performed for the 5 signature proteins. After normalization, the expression levels of the 5 proteins would be scaled as equation (1). The upper and lower for each protein are listed in Table S4.
The probability of each patient would be calculated as:

$$prob(x) = \frac{1}{1 + \exp(\text{-}0.313025 \cdot f(x)\text{-}0.239983)}, \tag{4}$$

where $f(x)$ is calculated as:

$$f(x) = \sum_{i=1}^{47} sv\_coef(i) \cdot \exp(-0.5 \cdot \|SVs(i) - x\|^2) + 0.195726 . \quad (5)$$

In this equation, $SVs(i)$ and $sv\_coef(i)$ are support vectors and related coefficients (Table S5).

When the probability calculated is smaller than the preset threshold of 0.597, the patient is classified into poor-prognosis group; otherwise, the patient is classified into good-prognosis group.

References

1. Feng M, Gao W, Wang R, Chen W, Man Y-G, Figg WD, et al. Therapeutically targeting glypican-3 via a conformation-specific single-domain antibody in hepatocellular carcinoma. Proc. Natl. Acad. Sci. 2013;110:E1083–91.

2. Chen M, Pratt CP, Zeeman ME, Schultz N, Taylor BS, O'Neill A, et al. Identification of PHLPP1 as a tumor suppressor reveals the role of feedback activation in PTEN-mutant prostate cancer progression. Cancer Cell. 2011;20:173–86.

3. Healy JE, Gearhart CN, Bateman JL, Handa RJ, Florant GL. AMPK and ACC change with fasting and physiological condition in euthermic and hibernating golden-mantled ground squirrels (Callospermophilus lateralis). Comp. Biochem. Physiol. A. Mol. Integr. Physiol. 2011;159:322–31.

4. Pannone G, Hindi S a. H, Santoro A, Sanguedolce F, Rubini C, Cincione RI, et al. Aurora B expression as a prognostic indicator and possible therapeutic target in oral squamous cell carcinoma. Int. J. Immunopathol. Pharmacol. 2011;24:79–88.

5. Mino-Kenudson M, Chirieac LR, Law K, Hornick JL, Lindeman N, Mark EJ, et al. A novel, highly sensitive antibody allows for the routine detection of ALK-rearranged lung adenocarcinomas by standard immunohistochemistry. Clin. Cancer Res. Off. J. Am. Assoc. Cancer Res. 2010;16:1561–71.

6. Ren H, Tan Z-P, Zhu X, Crosby K, Haack H, Ren J-M, et al. Identification of anaplastic lymphoma kinase as a potential therapeutic target in ovarian cancer. Cancer Res. 2012;72:3312–23.

7. Behnsawy HM, Miyake H, Abdalla MA, Sayed MA, Ahmed AE-FI, Fujisawa M. Expression of cell cycle-associated proteins in non-muscle-invasive bladder cancer: correlation with intravesical recurrence following transurethral resection. Urol. Oncol. 2011;29:495–501.

8. Weisberg E, Ray A, Nelson E, Adamia S, Barrett R, Sattler M, et al. Reversible resistance induced by FLT3 inhibition: a novel resistance mechanism in mutant FLT3-expressing cells. PloS One. 2011;6:e25351.

9. Schuetz JM, Johnson NA, Morin RD, Scott DW, Tan K, Ben-Nierah S, et al. BCL2 mutations in diffuse large B-cell lymphoma. Leukemia. 2012;26:1383–90.

10. Bellas C, García D, Vicente Y, Kilany L, Abraira V, Navarro B, et al. Immunohistochemical and molecular characteristics with prognostic significance in diffuse large B-cell lymphoma. PloS One. 2014;9:e98169.

11. Némati F, de Montrion C, Lang G, Kraus-Berthier L, Carita G, Sastre-Garau X, et al. Targeting Bcl-2/Bcl-XL induces antitumor activity in uveal melanoma patient-derived xenografts. PloS One. 2014;9:e80836.

12. Cigna N, Farrokhi Moshai E, Brayer S, Marchal-Somme J, Wémeau-Stervinou L, Fabre A, et al. The hedgehog system machinery controls transforming growth factor-β-dependent myofibroblastic differentiation in humans: involvement in idiopathic pulmonary fibrosis. Am. J. Pathol. 2012;181:2126–37.

13. Zinn RL, Gardner EE, Dobromilskaya I, Murphy S, Marchionni L, Hann CL, et al. Combination treatment with ABT-737 and chloroquine in preclinical models of small cell lung cancer. Mol. Cancer. 2013;12:16.

14. Barbone D, Ryan JA, Kolhatkar N, Chacko AD, Jablons DM, Sugarbaker DJ, et al. The Bcl-2 repertoire of mesothelioma spheroids underlies acquired apoptotic multicellular resistance. Cell Death Dis. 2011;2:e174.

15. Trautmann M, Sievers E, Aretz S, Kindler D, Michels S, Friedrichs N, et al. SS18-SSX fusion protein-induced Wnt/β-catenin signaling is a therapeutic target in synovial sarcoma. Oncogene. 2013; 33, 5006-5016

16. Abdi J, Mutis T, Garssen J, Redegeld F. Stimulation of Toll-like receptor-1/2 combined with Velcade increases cytotoxicity to human multiple myeloma cells. Blood Cancer J. 2013;3:e119.

17. Stutz N, Johnson RD, Wood GS. The Fas apoptotic pathway in cutaneous T-cell lymphomas: frequent expression of phenotypes associated with resistance to apoptosis. J. Am. Acad. Dermatol. 2012;67:1327.e1-10.

18. Hu K, Liao D, Wu W, Han A-J, Shi H-J, Wang F, et al. Targeting the anaphase-promoting complex/cyclosome (APC/C)- bromodomain containing 7 (BRD7) pathway for human osteosarcoma. Oncotarget. 2014;5:3088–100.

19. Palmer A, Gavin AC, Nebreda AR. A link between MAP kinase and p34(cdc2)/cyclin B during oocyte maturation: p90(rsk) phosphorylates and inactivates the p34(cdc2) inhibitory kinase Myt1. EMBO J. 1998;17:5037–47.

20. Ye XS, Fincher RR, Tang A, McNeal KK, Gygax SE, Wexler AN, et al. Proteolysis and tyrosine phosphorylation of p34cdc2/cyclin B. The role of MCM2 and initiation of DNA replication to allow tyrosine phosphorylation of p34cdc2. J. Biol. Chem. 1997;272:33384–93.

21. Leich E, Zamo A, Horn H, Haralambieva E, Puppe B, Gascoyne RD, et al. MicroRNA profiles of t(14;18)-negative follicular lymphoma support a late germinal center B-cell phenotype. Blood. 2011;118:5550–8.

22. Ehlen O, Nodin B, Rexhepaj E, Brandstedt J, Uhlen M, Alvarado-Kristensson M, et al. RBM3-Regulated Genes Promote DNA Integrity and Affect Clinical Outcome in Epithelial Ovarian Cancer. Transl. Oncol. 2011;4:212–21.

23. Wong NACS, Melegh Z. Antigen retrieval and primary antibody type affect sensitivity but not specificity of CD117 immunohistochemistry. Histopathology. 2009;54:529–38.

24. Rosenbaum ST, Svalø J, Nielsen K, Larsen T, Jørgensen JC, Bouchelouche P. Immunolocalization and expression of small-conductance calcium-activated potassium channels in human myometrium. J. Cell. Mol. Med. 2012;16:3001–8.

25. Jacobsen F, Ashtiani SN, Tennstedt P, Heinzer H, Simon R, Sauter G, et al. High c-MET expression is frequent but not associated with early PSA recurrence in prostate cancer. Exp. Ther. Med. 2013;5:102–6.

26. Terwisscha van Scheltinga AGT, Lub-de Hooge MN, Hinner MJ, Verheijen RB, Allersdorfer A, Hülsmeyer M, et al. In vivo visualization of MET tumor expression and anticalin biodistribution with the MET-specific anticalin 89Zr-PRS-110 PET tracer. J. Nucl. Med. Off. Publ. Soc. Nucl. Med. 2014;55:665–71.

27. Ohe M, Yokose T, Sakuma Y, Miyagi Y, Okamoto N, Osanai S, et al. Stromal micropapillary component as a novel unfavorable prognostic factor of lung adenocarcinoma. Diagn. Pathol. 2012;7:3.

28. Toon CW, Chou A, Clarkson A, DeSilva K, Houang M, Chan JCY, et al. Immunohistochemistry for myc predicts survival in colorectal cancer. PloS One. 2014;9:e87456.

29. Sanz-Moreno A, Fuhrmann D, Wolf E, von Eyss B, Eilers M, Elsässer H-P. Miz1 deficiency in the mammary gland causes a lactation defect by attenuated Stat5 expression and phosphorylation. PloS One. 2014;9:e89187.

30. Hu S, Xu-Monette ZY, Tzankov A, Green T, Wu L, Balasubramanyam A, et al. MYC/BCL2 protein coexpression contributes to the inferior survival of activated B-cell subtype of diffuse large B-cell lymphoma and demonstrates high-risk gene expression signatures: a report from The International DLBCL Rituximab-CHOP Consortium Program. Blood. 2013;121:4021–4031; quiz 4250.

31. Al-Salihi MA, Pearman AT, Doan T, Reichert EC, Rosenberg DW, Prescott SM, et al. Transgenic expression of Cyclooxygenase-2 in mouse intestine epithelium is insufficient to initiate tumorigenesis but promotes tumor progression. Cancer Lett. 2009;273:225–32.

32. Tryfonopoulos D, Walsh S, Collins DM, Flanagan L, Quinn C, Corkery B, et al. Src: a potential target for the treatment of triple-negative breast cancer. Ann. Oncol. Off. J. Eur. Soc. Med. Oncol. ESMO. 2011;22:2234–40.

33. Pan X, Zhou T, Tai Y-H, Wang C, Zhao J, Cao Y, et al. Elevated expression of CUEDC2 protein confers endocrine resistance in breast cancer. Nat. Med. 2011;17:708–14.

34. Wheat R, Roberts C, Waterboer T, Steele J, Marsden J, Steven NM, et al. Inflammatory cell distribution in primary merkel cell carcinoma. Cancers. 2014;6:1047–64.

35. Feng Z, Guo W, Zhang C, Xu Q, Zhang P, Sun J, et al. CCND1 as a predictive biomarker of neoadjuvant chemotherapy in patients with locally advanced head and neck squamous cell carcinoma. PloS One. 2011;6:e26399.

36. Zhong L-P, Zhu D-W, William WN, Liu Y, Ma J, Yang C-Z, et al. Elevated cyclin D1 expression is predictive for a benefit from TPF induction chemotherapy in oral squamous cell carcinoma patients with advanced nodal disease. Mol. Cancer Ther. 2013;12:1112–21.

37. Kuhn E, Bahadirli-Talbott A, Shih I-M. Frequent CCNE1 amplification in endometrial intraepithelial carcinoma and uterine serous carcinoma. Mod. Pathol. Off. J. U. S. Can. Acad. Pathol. Inc. 2014;27:1014–9.

38. Roudier M, Coleman I, Zhang X, Coleman R, Chery L, Brown L, et al. Abstract 5122: Characterizing the molecular features of ERG positive tumors in primary and castration resistant prostate cancer. Cancer Res. 2013;73:5122–5122.

39. Gross O, Beirowski B, Harvey SJ, Mcfadden C, Chen D, Tam S, et al. DDR1-deficient mice show localized

subepithelial GBM thickening with focal loss of slit diaphragms and proteinuria. Kidney Int. 2004;66:102–11.

40. Turashvili G, Bouchal J, Ehrmann J, Fridman E, Skarda J, Kolar Z. Novel immunohistochemical markers for the differentiation of lobular and ductal invasive breast carcinomas. Biomed. Pap. Med. Fac. Univ. Palacký Olomouc Czechoslov. 2007;151:59–64.

41. Reichert-Faria A, Jung J e., Moreschi Neto V, Silva de Castro C c., Mira M t., Noronha L. Reduced immunohistochemical expression of Discoidin Domain Receptor 1 (DDR1) in vitiligo skin. J. Eur. Acad. Dermatol. Venereol. 2013;27:1057–9.

42. DNA-PK, the DNA-activated protein kinase, is differentially expressed in normal and malignant human tissues. Publ. Online 20 May 1999 Doi101038sjonc1202640 [Internet]. 1999 [cited 2014 Sep 11];18. Available from: http://www.nature.com/onc/journal/v18/n20/full/1202640a.html

43. Björk-Eriksson T, West C, Nilsson A, Magnusson B, Svensson M, Karlsson E, et al. The immunohistochemical expression of DNA-PKCS and Ku (p70/p80) in head and neck cancers: relationships with radiosensitivity. Int. J. Radiat. Oncol. Biol. Phys. 1999;45:1005–10.

44. Liu M, Casimiro MC, Wang C, Shirley LA, Jiao X, Katiyar S, et al. p21CIP1 attenuates Ras- and c-Myc-dependent breast tumor epithelial mesenchymal transition and cancer stem cell-like gene expression in vivo. Proc. Natl. Acad. Sci. U. S. A. 2009;106:19035–9.

45. Kotb AM, Hierholzer A, Kemler R. Replacement of E-cadherin by N-cadherin in the mammary gland leads to fibrocystic changes and tumor formation. Breast Cancer Res. BCR. 2011;13:R104.

46. Haro E, Delgado I, Junco M, Yamada Y, Mansouri A, Oberg KC, et al. Sp6 and Sp8 Transcription Factors Control AER Formation and Dorsal-Ventral Patterning in Limb Development. PLoS Genet. 2014;10:e1004468.

47. Frederick MJ, VanMeter AJ, Gadhikar MA, Henderson YC, Yao H, Pickering CC, et al. Phosphoproteomic analysis of signaling pathways in head and neck squamous cell carcinoma patient samples. Am. J. Pathol. 2011;178:548–71.

48. Modena P, Buttarelli FR, Miceli R, Piccinin E, Baldi C, Antonelli M, et al. Predictors of outcome in an AIEOP series of childhood ependymomas: a multifactorial analysis. Neuro-Oncol. 2012;14:1346–56.

49. Kato Y, Peled N, Wynes MW, Yoshida K, Pardo M, Mascaux C, et al. Novel epidermal growth factor receptor mutation-specific antibodies for non-small cell lung cancer: immunohistochemistry as a possible screening method for epidermal growth factor receptor mutations. J. Thorac. Oncol. Off. Publ. Int. Assoc. Study Lung Cancer. 2010;5:1551–8.

50. Kato Y, Peled N, Wynes MW, Yoshida K, Pardo M, Mascaux C, et al. Novel epidermal growth factor receptor mutation-specific antibodies for non-small cell lung cancer: immunohistochemistry as a possible screening method for epidermal growth factor receptor mutations. J. Thorac. Oncol. Off. Publ. Int. Assoc. Study Lung Cancer. 2010;5:1551–8.

51. Behnsawy HM, Miyake H, Abdalla MA, Sayed MA, Ahmed AE-FI, Fujisawa M. Expression of cell cycle-associated proteins in non-muscle-invasive bladder cancer: correlation with intravesical recurrence following transurethral resection. Urol. Oncol. 2011;29:495–501.

52. Lin D-C, Hao J-J, Nagata Y, Xu L, Shang L, Meng X, et al. Genomic and molecular characterization of esophageal squamous cell carcinoma. Nat. Genet. 2014;46:467–73.

53. Zhang J, Wu G, Miller CP, Tatevossian RG, Dalton JD, Tang B, et al. Whole-genome sequencing identifies genetic alterations in pediatric low-grade gliomas. Nat. Genet. 2013;45:602–12.

54. Chatterjee A, Chatterjee U, Ghosh MK. Activation of protein kinase CK2 attenuates FOXO3a functioning in a PML-dependent manner: implications in human prostate cancer. Cell Death Dis. 2013;4:e543.

55. Gobrecht P, Leibinger M, Andreadaki A, Fischer D. Sustained GSK3 activity markedly facilitates nerve regeneration. Nat. Commun. [Internet]. 2014 [cited 2014 Oct 9];5. Available from: http://www.nature.com/ncomms/2014/140731/ncomms5561/full/ncomms5561.html

56. Colón-Díaz M, Báez-Vega P, García M, Ruiz A, Monteiro JB, Fourquet J, et al. HDAC1 and HDAC2 are differentially expressed in endometriosis. Reprod. Sci. Thousand Oaks Calif. 2012;19:483–92.

57. Trivedi CM, Luo Y, Yin Z, Zhang M, Zhu W, Wang T, et al. Hdac2 regulates the cardiac hypertrophic response by modulating Gsk3β activity. Nat. Med. 2007;13:324–31.

58. Wilson AJ, Byun D-S, Popova N, Murray LB, L'Italien K, Sowa Y, et al. Histone Deacetylase 3 (HDAC3) and Other Class I HDACs Regulate Colon Cell Maturation and p21 Expression and Are Deregulated in Human Colon Cancer. J. Biol. Chem. 2006;281:13548–58.

59. Minamiya Y, Ono T, Saito H, Takahashi N, Ito M, Motoyama S, et al. Strong expression of HDAC3 correlates with a poor prognosis in patients with adenocarcinoma of the lung. Tumor Biol. 2010;31:533–9.

60. Xiao H, Tong R, Cheng S, Lv Z, Ding C, Du C, et al. BAG3 and HIF-1 α coexpression detected by immunohistochemistry correlated with prognosis in hepatocellular carcinoma after liver transplantation. BioMed Res. Int. 2014;2014:516518.

61. Zeindl-Eberhart E, Brandl L, Liebmann S, Ormanns S, Scheel SK, Brabletz T, et al. Epithelial-mesenchymal transition induces endoplasmic-reticulum-stress response in human colorectal tumor cells. PloS One. 2014;9:e87386.

62. Chen L, Shi Y, Yuan J, Han Y, Qin R, Wu Q, et al. HIF-1 alpha overexpression correlates with poor overall survival and disease-free survival in gastric cancer patients post-gastrectomy. PloS One. 2014;9:e90678.

63. Sebastiá R, Ventura MP, Solari HP, Antecka E, Orellana ME, Burnier MN. Immunohistochemical detection of Hsp90 and Ki-67 in pterygium. Diagn. Pathol. 2013;8:32.

64. Sebastiá R, Ventura MP, Solari HP, Antecka E, Orellana ME, Jr MNB. Immunohistochemical detection of Hsp90 and Ki-67 in pterygium. Diagn. Pathol. 2013;8:1–7.

65. Pieper M, Rupprecht HD, Bruch KM, De Heer E, Schöcklmann HO. Requirement of heat shock protein 90 in mesangial cell mitogenesis. Kidney Int. 2000;58:2377–89.

66. Vivanco I, Palaskas N, Tran C, Finn SP, Getz G, Kennedy NJ, et al. Identification of the JNK Signaling Pathway as a Functional Target of the Tumor Suppressor PTEN. Cancer Cell. 2007;11:555–69.

67. Savage MJ, Lin Y-G, Ciallella JR, Flood DG, Scott RW. Activation of c-Jun N-terminal kinase and p38 in an Alzheimer's disease model is associated with amyloid deposition. J. Neurosci. Off. J. Soc. Neurosci. 2002;22:3376–85.

68. Gonzalo S, Grasa L, Hernández LV, Arruebo MP, Plaza MÁ, Murillo MD. Mitogen activated protein kinases blockade improves lipopolysaccharide-induced ileal motor disturbances. Rev. Esp. Enfermedades Dig. Organo Of. Soc. Esp. Patol. Dig. 2012;104:305–9.

69. Byrne R, Rath E, Hladik A, Niederreiter B, Bonelli M, Frantal S, et al. A dynamic real time in vivo and static ex vivo analysis of granulomonocytic cell migration in the collagen-induced arthritis model. PloS One. 2012;7:e35194.

70. Kirabo A, Park SO, Majumder A, Gali M, Reinhard MK, Wamsley HL, et al. The Jak2 inhibitor, G6, alleviates Jak2-V617F-mediated myeloproliferative neoplasia by providing significant therapeutic efficacy to the bone marrow. Neoplasia N. Y. N. 2011;13:1058–68.

71. Nakada Y, Stewart TG, Peña CG, Zhang S, Zhao N, Bardeesy N, et al. The LKB1 tumor suppressor as a biomarker in mouse and human tissues. PloS One. 2013;8:e73449.

72. Kong L, Zhang G, Wang X, Zhou J, Hou S, Cui W. Immunohistochemical expression of RBP2 and LSD1 in papillary thyroid carcinoma. Romanian J. Morphol. Embryol. Rev. Roum. Morphol. Embryol. 2013;54:499–503.

73. Metzger E, Wissmann M, Yin N, Müller JM, Schneider R, Peters AHFM, et al. LSD1 demethylates repressive histone marks to promote androgen-receptor-dependent transcription. Nature. 2005;437:436–9.

74. Wu Y, Wang Y, Yang XH, Kang T, Zhao Y, Wang C, et al. The Deubiquitinase USP28 Stabilizes LSD1 and Confers Stem-Cell-like Traits to Breast Cancer Cells. Cell Rep. 2013;5:224–36.

75. Furlong F, Fitzpatrick P, O'Toole S, Phelan S, McGrogan B, Maguire A, et al. Low MAD2 expression levels associate with reduced progression-free survival in patients with high-grade serous epithelial ovarian cancer. J. Pathol. 2012;226:746–55.

76. Park PE, Jeong JY, Kim SZ, Park JY. MAD2 Expression in Ovarian Carcinoma: Different Expression Patterns and

Levels among Various Types of Ovarian Carcinoma and Its Prognostic Significance in High-Grade Serous Carcinoma. Korean J. Pathol. 2013;47:418.

77. Kato T, Daigo Y, Aragaki M, Ishikawa K, Sato M, Kondo S, et al. Overexpression of MAD2 predicts clinical outcome in primary lung cancer patients. Lung Cancer. 2011;74:124–31.

78. Aleixo PB, Hartmann AA, Menezes IC, Meurer RT, Oliveira AM. Can MDM2 and CDK4 make the diagnosis of well differentiated/dedifferentiated liposarcoma? An immunohistochemical study on 129 soft tissue tumours. J. Clin. Pathol. 2009;62:1127–35.

79. Thway K, Flora R, Shah C, Olmos D, Fisher C. Diagnostic utility of p16, CDK4, and MDM2 as an immunohistochemical panel in distinguishing well-differentiated and dedifferentiated liposarcomas from other adipocytic tumors. Am. J. Surg. Pathol. 2012;36:462–9.

80. Dujardin F, Binh MBN, Bouvier C, Gomez-Brouchet A, Larousserie F, Muret A de, et al. MDM2 and CDK4 immunohistochemistry is a valuable tool in the differential diagnosis of low-grade osteosarcomas and other primary fibro-osseous lesions of the bone. Mod. Pathol. 2011;24:624–37.

81. Yu G, Wang J, Chen Y, Wang X, Pan J, Li G, et al. Overexpression of phosphorylated mammalian target of rapamycin predicts lymph node metastasis and prognosis of chinese patients with gastric cancer. Clin. Cancer Res. Off. J. Am. Assoc. Cancer Res. 2009;15:1821–9.

82. Sakamoto K, Morita K-I, Shimada Y, Omura K, Izumo T, Yamaguchi A. Peripheral odontogenic keratocyst associated with nevoid basal cell carcinoma syndrome: a case report. Oral Surg. Oral Med. Oral Pathol. Oral Radiol. 2014;118:e19-23.

83. Liu S-C, Tsang N-M, Chiang W-C, Chang K-P, Hsueh C, Liang Y, et al. Leukemia inhibitory factor promotes nasopharyngeal carcinoma progression and radioresistance. J. Clin. Invest. 2013;123:5269–83.

84. Souslova V, Townsend PA, Mann J, van der Loos CM, Motterle A, D'Acquisto F, et al. Allele-specific regulation of matrix metalloproteinase-3 gene by transcription factor NFkappaB. PloS One. 2010;5:e9902.

85. Li R, Ma T, Gu J, Liang X, Li S. Imbalanced network biomarkers for traditional Chinese medicine Syndrome in gastritis patients. Sci. Rep. 2013;3:1543.

86. Urashima M, Hama T, Suda T, Suzuki Y, Ikegami M, Sakanashi C, et al. Distinct effects of alcohol consumption and smoking on genetic alterations in head and neck carcinoma. PloS One. 2013;8:e80828.

87. Shan M, Zhang X, Liu X, Qin Y, Liu T, Liu Y, et al. P16 and p53 play distinct roles in different subtypes of breast cancer. PloS One. 2013;8:e76408.

88. Hou X, Li Y, Luo R-Z, Fu J-H, He J-H, Zhang L-J, et al. High expression of the transcriptional co-activator p300 predicts poor survival in resectable non-small cell lung cancers. Eur. J. Surg. Oncol. J. Eur. Soc. Surg. Oncol. Br. Assoc. Surg. Oncol. 2012;38:523–30.

89. Lemos JC, Roth CA, Messinger DI, Gill HK, Phillips PEM, Chavkin C. Repeated stress dysregulates κ-opioid receptor signaling in the dorsal raphe through a p38α MAPK-dependent mechanism. J. Neurosci. Off. J. Soc. Neurosci. 2012;32:12325–36.

90. Zhou G, Wang J, Zhao M, Xie T-X, Tanaka N, Sano D, et al. Gain-of-function mutant p53 promotes cell growth and cancer cell metabolism via inhibition of AMPK activation. Mol. Cell. 2014;54:960–74.

91. Bedelbaeva K, Snyder A, Gourevitch D, Clark L, Zhang X-M, Leferovich J, et al. Lack of p21 expression links cell cycle control and appendage regeneration in mice. Proc. Natl. Acad. Sci. U. S. A. 2010;107:5845–50.

92. Dong M, Wei H, Hou J-M, Gao S, Yang D-Z, Lin Z-H, et al. Possible prognostic significance of p53, cyclin D1 and Ki-67 in the second primary malignancy of patients with double primary malignancies. Int. J. Clin. Exp. Pathol. 2014;7:3975–83.

93. Evangelou K, Bartkova J, Kotsinas A, Pateras IS, Liontos M, Velimezi G, et al. The DNA damage checkpoint precedes activation of ARF in response to escalating oncogenic stress during tumorigenesis. Cell Death Differ. 2013;20:1485–97.

94. Lantuejoul S, Raynaud C, Salameire D, Gazzeri S, Moro-Sibilot D, Soria J-C, et al. Telomere maintenance and DNA

damage responses during lung carcinogenesis. Clin. Cancer Res. Off. J. Am. Assoc. Cancer Res. 2010;16:2979–88.

95. Lee S-H, Jung Y-S, Chung J-Y, Oh AY, Lee S-J, Choi DH, et al. Novel tumor suppressive function of Smad4 in serum starvation-induced cell death through PAK1-PUMA pathway. Cell Death Dis. 2011;2:e235.

96. Nupponen NN, Paulsson J, Jeibmann A, Wrede B, Tanner M, Wolff JEA, et al. Platelet-derived growth factor receptor expression and amplification in choroid plexus carcinomas. Mod. Pathol. Off. J. U. S. Can. Acad. Pathol. Inc. 2008;21:265–70.

97. Cui W, Cai Y, Wang W, Liu Z, Wei P, Bi R, et al. Frequent copy number variations of PI3K/AKT pathway and aberrant protein expressions of PI3K subunits are associated with inferior survival in diffuse large B cell lymphoma. J. Transl. Med. 2014;12:10.

98. Iyengar S, Clear A, Bödör C, Maharaj L, Lee A, Calaminici M, et al. P110α-mediated constitutive PI3K signaling limits the efficacy of p110δ-selective inhibition in mantle cell lymphoma, particularly with multiple relapse. Blood. 2013;121:2274–84.

99. Kanaji S, Saito H, Tsujitani S, Matsumoto S, Tatebe S, Kondo A, et al. Expression of polo-like kinase 1 (PLK1) protein predicts the survival of patients with gastric carcinoma. Oncology. 2006;70:126–33.

100. Weichert W, Kristiansen G, Schmidt M, Gekeler V, Noske A, Niesporek S, et al. Polo-like kinase 1 expression is a prognostic factor in human colon cancer. World J. Gastroenterol. WJG. 2005;11:5644–50.

101. Sha W, Thompson K, South J, Baron M, Leask A. Loss of PPARγ expression by fibroblasts enhances dermal wound closure. Fibrogenesis Tissue Repair. 2012;5:5.

102. Dumond H, Presle N, Pottie P, Pacquelet S, Terlain B, Netter P, et al. Site specific changes in gene expression and cartilage metabolism during early experimental osteoarthritis. Osteoarthr. Cartil. OARS Osteoarthr. Res. Soc. 2004;12:284–95.

103. Cheng H, Meng J, Wang G, Meng Y, Li Y, Wei D, et al. Skp2 regulates subcellular localization of PPARγ by MEK signaling pathways in human breast cancer. Int. J. Mol. Sci. 2013;14:16554–69.

104. Wullschleger S, Sakamoto K, Johnstone L, Duce S, Fleming S, Alessi DR. How moderate changes in Akt T-loop phosphorylation impact on tumorigenesis and insulin resistance. Dis. Model. Mech. 2011;4:95–103.

105. Saborowski A, Saborowski M, Davare MA, Druker BJ, Klimstra DS, Lowe SW. Mouse model of intrahepatic cholangiocarcinoma validates FIG-ROS as a potent fusion oncogene and therapeutic target. Proc. Natl. Acad. Sci. U. S. A. 2013;110:19513–8.

106. Miething C, Scuoppo C, Bosbach B, Appelmann I, Nakitandwe J, Ma J, et al. PTEN action in leukaemia dictated by the tissue microenvironment. Nature. 2014;510:402–6.

107. Li Y, Dowbenko D, Lasky LA. AKT/PKB phosphorylation of p21Cip/WAF1 enhances protein stability of p21Cip/WAF1 and promotes cell survival. J. Biol. Chem. 2002;277:11352–61.

108. Hodeify R, Tarcsafalvi A, Megyesi J, Safirstein RL, Price PM. Cdk2-dependent phosphorylation of p21 regulates the role of Cdk2 in cisplatin cytotoxicity. Am. J. Physiol. - Ren. Physiol. 2011;300:F1171–9.

109. Shin YJ, Kim Y-B, Kim J-H. Protein kinase CK2 phosphorylates and activates p21-activated kinase 1. Mol. Biol. Cell. 2013;24:2990–9.

110. Lyustikman Y, Momota H, Pao W, Holland EC. Constitutive activation of Raf-1 induces glioma formation in mice. Neoplasia N. Y. N. 2008;10:501–10.

111. Nagao S, Yamaguchi T, Kusaka M, Maser RL, Takahashi H, Cowley BD, et al. Renal activation of extracellular signal-regulated kinase in rats with autosomal-dominant polycystic kidney disease. Kidney Int. 2003;63:427–37.

112. Osorio FG, Bárcena C, Soria-Valles C, Ramsay AJ, de Carlos F, Cobo J, et al. Nuclear lamina defects cause ATM-dependent NF-κB activation and link accelerated aging to a systemic inflammatory response. Genes Dev. 2012;26:2311–24.

113. Qu X, Shen L, Zheng Y, Cui Y, Feng Z, Liu F, et al. A signal transduction pathway from TGF-β1 to SKP2 via Akt1 and c-Myc and its correlation with progression in human melanoma. J. Invest. Dermatol. 2014;134:159–67.

114. Liu J, Wei X-L, Huang W-H, Chen C-F, Bai J-W, Zhang G-J. Cytoplasmic Skp2 expression is associated with p-Akt1 and predicts poor prognosis in human breast carcinomas. PloS One. 2012;7:e52675.

115. Bao W, Wang H-H, Tian F-J, He X-Y, Qiu M-T, Wang J-Y, et al. A TrkB-STAT3-miR-204-5p regulatory circuitry controls proliferation and invasion of endometrial carcinoma cells. Mol. Cancer. 2013;12:155.

116. Wu J, Patmore DM, Jousma E, Eaves DW, Breving K, Patel AV, et al. EGFR-STAT3 signaling promotes formation of malignant peripheral nerve sheath tumors. Oncogene. 2014;33:173–80.

117. North PE, Waner M, Mizeracki A, Mihm MC. GLUT1: a newly discovered immunohistochemical marker for juvenile hemangiomas. Hum. Pathol. 2000;31:11–22.

118. Rakheja D, Khokhar S, Mitui M, Cost NG. Immunohistochemical expression of GLUT1 and its correlation with unfavorable histology and TP53 codon 72 polymorphism in Wilms tumors. Pediatr. Dev. Pathol. Off. J. Soc. Pediatr. Pathol. Paediatr. Pathol. Soc. 2012;15:286–92.

119. Kleeff J, Friess H, Simon P, Susmallian S, Buchler P, Zimmermann A, et al. Overexpression of Smad2 and Colocalization with TGF-β1 in Human Pancreatic Cancer. Dig. Dis. Sci. 1999;44:1793–802.

120. Annovazzi L, Mellai M, Caldera V, Valente G, Schiffer D. SOX2 Expression and Amplification in Gliomas and Glioma Cell Lines. Cancer Genomics - Proteomics. 2011;8:139–47.

121. Sholl LM, Long KB, Hornick JL. Sox2 expression in pulmonary non-small cell and neuroendocrine carcinomas. Appl. Immunohistochem. Mol. Morphol. AIMM Off. Publ. Soc. Appl. Immunohistochem. 2010;18:55–61.

122. Hussenet T, Dali S, Exinger J, Monga B, Jost B, Dembelé D, et al. SOX2 Is an Oncogene Activated by Recurrent 3q26.3 Amplifications in Human Lung Squamous Cell Carcinomas. PLoS ONE. 2010;5:e8960.

123. Li F, Cheng Q, Ling X, Stablewski A, Tang L, Foster BA, et al. Generation of a novel transgenic mouse model for bioluminescent monitoring of survivin gene activity in vivo at various pathophysiological processes: survivin expression overlaps with stem cell markers. Am. J. Pathol. 2010;176:1629–38.

124. Bollrath J, Phesse TJ, von Burstin VA, Putoczki T, Bennecke M, Bateman T, et al. gp130-mediated Stat3 activation in enterocytes regulates cell survival and cell-cycle progression during colitis-associated tumorigenesis. Cancer Cell. 2009;15:91–102.

125. Han X, Li F, Fang Z, Gao Y, Li F, Fang R, et al. Transdifferentiation of lung adenocarcinoma in mice with Lkb1 deficiency to squamous cell carcinoma. Nat. Commun. 2014;5:3261.

126. Parris TZ, Kovács A, Aziz L, Hajizadeh S, Nemes S, Semaan M, et al. Additive effect of the AZGP1, PIP, S100A8 and UBE2C molecular biomarkers improves outcome prediction in breast carcinoma. Int. J. Cancer J. Int. Cancer. 2014;134:1617–29.

127. Vecchione A, Belletti B, Lovat F, Volinia S, Chiappetta G, Giglio S, et al. A microRNA signature defines chemoresistance in ovarian cancer through modulation of angiogenesis. Proc. Natl. Acad. Sci. U. S. A. 2013;110:9845–50.
